# Woody flora of the Prof. Dr. Karl Arens Reserve, Corumbataí, São Paulo, Brazil

**DOI:** 10.3897/BDJ.13.e142217

**Published:** 2025-02-03

**Authors:** Lilian Silva Santos, Pedro Luís Rodrigues de Moraes

**Affiliations:** 1 Universidade Estadual Paulista (UNESP), Instituto de Biociências de Rio Claro, Programa de Pós-Graduação em Biologia Vegetal (Interunidades), Rio Claro, Brazil Universidade Estadual Paulista (UNESP), Instituto de Biociências de Rio Claro, Programa de Pós-Graduação em Biologia Vegetal (Interunidades) Rio Claro Brazil; 2 Universidade Estadual Paulista (UNESP), Instituto de Biociências de Rio Claro, Departamento de Biodiversidade, Rio Claro, Brazil, Rio Claro, Brazil Universidade Estadual Paulista (UNESP), Instituto de Biociências de Rio Claro, Departamento de Biodiversidade, Rio Claro, Brazil Rio Claro Brazil

**Keywords:** *Cerradão*, *Cerrado*, floristics, herbarium, species

## Abstract

**Background:**

This study was conducted in the Prof. Dr. Karl Arens Reserve, Corumbataí, São Paulo. The area is a *Cerrado* fragment, with no records of fire since 1962 and is characterised by the predominance of *Cerradão* phytophysiognomy. Although several studies have been conducted in the Reserve, there was no testimony material for some species in the Herbarium Rioclarense (HRCB), nor a list of vouchers for any taxon in the main publications. Thus, the objective of this work was to undertake a floristic survey of the woody species in the Reserve, based on herbarium specimens and fieldwork.

**New information:**

The survey in HRCB recorded 510 exsiccatae belonging to 160 species and 44 families. We also conducted 24 fieldwork expeditions between 2019 and 2021 and recorded 118 species in 39 families. In total, 193 woody species were recorded for the Reserve in Corumbataí. Species such as *Copaiferalangsdorffii* and *Myrcianeoclusiifolia* had already been cited for the area, but there was no testimony material in the herbarium. *Machaeriumnyctitans* and *Alchorneaglandulosa* are examples of new records for the Reserve. Fabaceae, Asteraceae, Myrtaceae, Melastomataceae and Rubiaceae were the families with the greatest species diversity.

## Introduction

The Prof. Dr. Karl Arens Reserve was obtained in 1962 by the São Paulo Research Foundation (Fundação de Amparo à Pesquisa do Estado de São Paulo - FAPESP) and is currently managed by the Institute of Bioscience, Rio Claro, of the São Paulo State University (UNESP). It is a fragment of the *Cerrado*, isolated by sugarcane plantations and cattle pastures and has no records of fire since its acquirement (Camargo and Arens 1967, Piccolo et al. 1971).

The *Cerrado* is the second largest phytogeographic domain in Brazil (Ab'Sáber 1983, Batalha 2011) and occupies 23% of the country (Ribeiro and Walter 2008). This important hotspot (Myers et al. 2000, Strassburg et al. 2017) represents 30% of the biodiversity in the country (Françoso et al. 2015). In the State of São Paulo, the *Cerrado* vegetation is severely fragmented (Eiten 1994, Cassavan 2002, Nalon et al. 2022), being part of the most threatened *Cerrado* ecoregion (Sano et al. 2019). According to the last inventory of the native vegetation cover (Nalon et al. 2022), of the 8,106,085 ha of original cover, only 239,312 ha remain.

The vegetation in the *Cerrado* is not uniform (Coutinho 2002) and includes field, savannah and forest physiognomies (Ribeiro and Walter 2008). Fire can change *Cerrado* physiognomies, impacting the vegetation (Eiten 1972). The absence of fire allows a savannah formation to become denser and form *Cerradão*, which is already the dominant formation in São Paulo State (Durigan et al. 2004a).

*Cerradão* is the tallest physiognomy in the *Cerrado* (Eiten 1994), characterised by a forest formation with trees 8–15 m tall, mostly continuous canopy and distinct layers of shrubs and herbs in the understorey due to variation in light reaching the ground. Individuals that occur in *cerrado* *sensu stricto* and other forest formations are common in this physiognomy (Ribeiro and Walter 2008). The Reserve in Corumbataí comprises *Cerradão* and *Cerrado* *sensu stricto* (Pinheiro et al. 2010, Pinheiro et al. 2021). The change in the proportion of the area occupied by each physiognomy is notable over more than 60 years. Due to the expansion and dominance of *Cerradão* in the fragment, only 3% of the *Cerrado* *sensu stricto* remains out of more than 34 initial hectares (Pinheiro et al. 2021). This phenomenon is mainly associated with the absence of fire (Pinheiro et al. 2010, Pinheiro et al. 2021), which changes the landscape, provokes a decrease in biodiversity and mostly harms non-arboreal species (Durigan 2020).

Since the Institute of Biosciences at UNESP in Rio Claro obtained custody of the Reserve, studies have been conducted in different areas of plant biology, mainly phytosociological and floristic works (Camargo and Arens 1967, Piccolo et al. 1971, Cesar et al. 1988, Campos 1989, Pagano et al. 1989, Paula and Monteiro 2000, Andena et al. 2005, Pinheiro 2013, Pinheiro et al. 2021).

Despite the studies and large species lists made for the Reserve, gaps were observed, such as the absence of testimony material of several taxa in the Herbarium Rioclarense (HRCB) and lack of citations of vouchers in those works. Thus, the objective of the present study was to identify and record the woody species in the Prof. Dr. Karl Arens Reserve, Corumbataí, São Paulo, based on a floristic survey and herbarium specimens mainly deposited at HRCB and from a set housed at SPFR, with images available in speciesLink (2024).

## Materials and methods

### Study area

**Figure 1.**
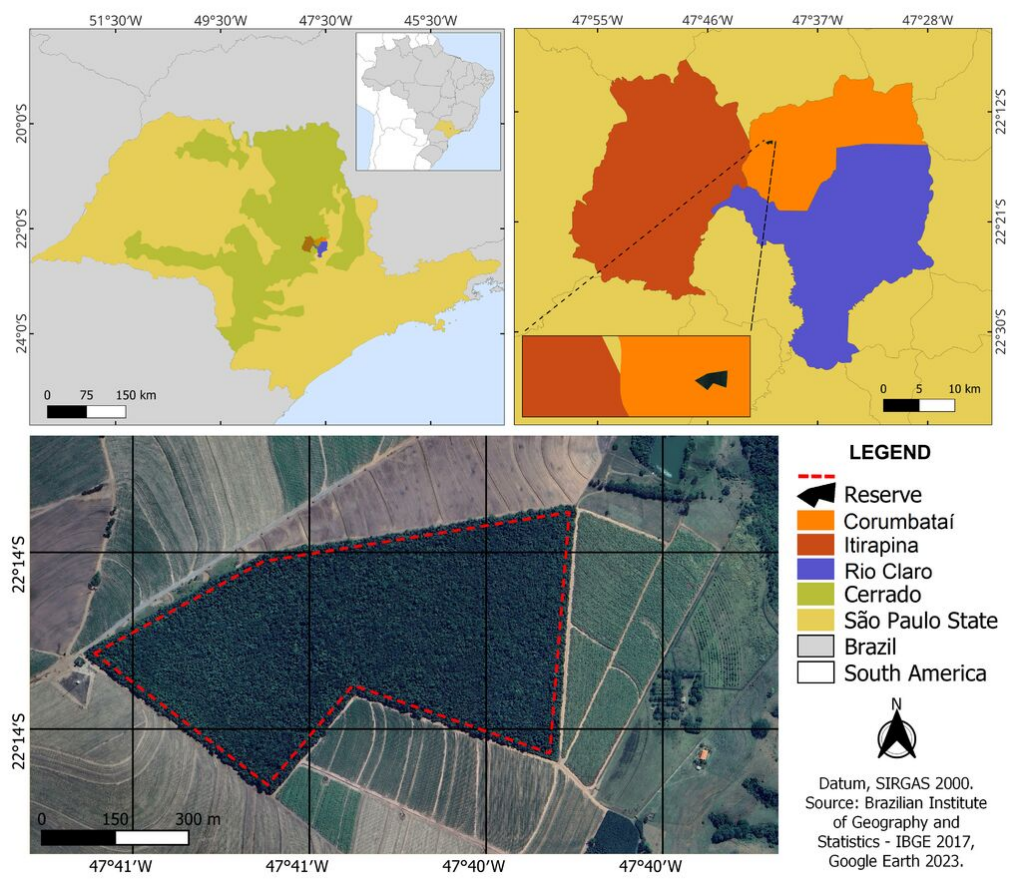
Study Area: location and natural coverage of *Cerrado* in São Paulo State, including the Municipality of Corumbataí, its border with Itirapina and Rio Claro and limits from Prof. Dr. Karl Arens Reserve. Prepared by: Lilian S. Santos.

The Prof. Dr. Karl Arens Reserve (22°14'30"S, 47°41'10"W) is located in Corumbataí, São Paulo, approximately 30 km from the city of Rio Claro (Fig. 1) and 200 km from the state capital. The study area is in the Paraná Sedimentary Basin, in a transition region between the Basalt Cuestas and Peripheral Depression geomorphological Provinces (Troppmair 2000, Marques e Amorim et al. 2005), more specifically on the Residual Plateau of Brotas-Itirapina (IBGE 2023).

The fragment is in the *Cerrado* domain and has an area of almost 39 ha (Pinheiro et al. 2010). The area is surrounded by a monoculture of sugarcane and pasture and ranges from 793–866 m. *Cerradão* is predominant in relation to *Cerrado* *sensu strictu* (Pinheiro et al. 2021) and there are fragments of semi-deciduous seasonal forest on nearby hills (Pinheiro et al. 2010, Pinheiro et al. 2021).

The soil is a dystrophic, Red-Yellow/Red Latosol with a medium texture (Rossi 2017) and, despite the presence of clay, is predominantly sandy (Cesar et al. 1988, Pinheiro et al. 2010). The climate in the region is the Cwa type: subtropical humid with a cold and dry period between April and September and a hot and rainy period between October and March (Peel et al. 2007). The average temperature from 2015–2023 was 21.8ºC, with a minimum of 12.8ºC and a maximum of 30.9ºC (CIIAGRO 2023). The average annual precipitation was 1492 mm between 1987 and 2017 (ANA 2021) and 1197 mm between 2018 and 2023 (CIIAGRO 2023).

### Obtaining and analysing the material

Exsiccatae from the Reserve in Corumbataí were selected from the Herbarium Rioclarense (HRCB), which belongs to the Institute of Biosciences at UNESP in Rio Claro. HRCB has the largest and most important collection of specimens from the Reserve of Corumbataí, with duplicates that were distributed to many herbaria i.e.: ALCB, BHCB, CEN, CESJ, CPAP, ESA, FLOR, FUEL, HUEM, HUESB, HUFSJ, HUFU, HUPG, HXBH, IAC, INPA, MBM, MBML, MO, PAMG, PEUFR, SJRP, SP, SPF, SPFR, SPSF, UEC and UPCB. We manually checked all the fertile material with subshrub, shrub and tree habits in HRCB. We did not include herbs and vines because we lack adequate reference collections and we had limited time available for more extensive sampling efforts. Information, such as voucher number, identification, collector and collection date, were taken from the labels.

Additionally, we conducted rapid floristic walkover surveys on foot (Filgueiras et al. 1994) in the fragment to collect woody and subwoody material, except for vines. However, facultative climbers were considered in this study, as well as "woody" palms. During these field expeditions, we walked along the entire perimeter of the Reserve, along the trail that crosses the fragment, as well as into the middle of the fragment from this trail. There were no predetermined transects; the walking was random and focused on scanning as much area as possible in search of fertile material. A drone was used to take images of the area and we took photographs *in situ* from the specimens to be obtained. The collected material was processed in the herbarium (Fidalgo and Bononi 1989) and exsiccatae were prepared and incorporated into HRCB.

All the material obtained in the field and herbarium was checked and determined using studies conducted in the area (Camargo and Arens 1967, Piccolo et al. 1971, Cesar et al. 1988, Pagano et al. 1989, Andena et al. 2005, Pinheiro et al. 2010, Pinheiro 2013, Pinheiro et al. 2021), floras for the State, for example, Flora Fanerogâmica do Estado de São Paulo (Romão and Souza 2016) and field guides (Durigan et al. 2004b, Souza et al. 2018). The specimens were analysed using a Leica M80 stereomicroscope and the software Leica LAS EZ.

Morphological comparisons were made with material in HRCB and on virtual herbaria (e.g. MNHN (2023),speciesLink (2024)). Dictionaries and glossaries were used to help interpret the terminology and morphology (Font Quer 1953, Harris and Harris 1994, Hickey and King 2000, Gonçalves and Lorenzi 2011). Species life-form was verified in the Flora e Funga do Brasil (2024) platform and specialised literature of the different taxonomic groups. The families follow APG IV (2016), the epithets and authors were updated (IPNI 2024, speciesLink 2024) and the herbaria acronyms follow Thiers [continuously updated] (2024).

The software QGIS version 3.36.1 was used to create the maps and georeference the satellite image. The remote sensing image and coordinates were obtained with the software Google Earth Pro version 7.3.6. All figures were edited with the software Adobe Photoshop CS5.

## Data resources

**Figure 2.**
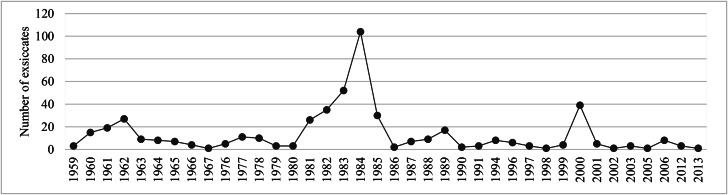
Specimens obtained from the HRCB collection: most collected periods in the Prof. Dr. Karl Arens Reserve.

We found 510 exsiccatae in HRCB, distributed in 160 species, 111 genera and 44 families of angiosperms (Table 1). Nearly half of the collections are from 1981–1985, especially 1984 with 104 exsiccatae (Fig. 2). During that period, most collections were gathered by O. Cesar & S.N. Pagano and M.J.O. Campos, resulting, respectively, in a publication on the phytosociological structure of the Reserve's tree stratum (Cesar et al. 1988) and a doctoral thesis (Campos 1989). Smaller collection peaks occurred in 1962, led by H. Amaral and, in 2000, with collections conducted by C.E. Carneiro, L. Cordeiro, V.B. Ziparro and by V.F.O. Miranda. However, the only work potentially associated with those collections is Piccolo et al. (1971). The complete list of the specimens analysed and their respective vouchers are in the Supplementary Material (Suppl. material 1).

**Figure 3.**
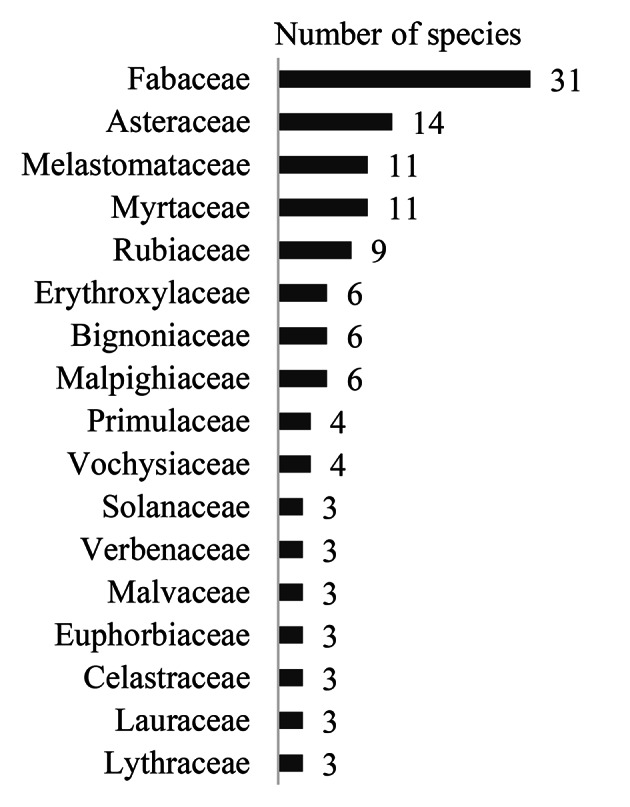
Specimens obtained from the HRCB collection: families with the largest diversity in species.

Fabaceae, Asteraceae, Melastomataceae, Myrtaceae and Rubiaceae were the families with the greatest species diversity in the HRCB collection (Fig. 3). Nevertheless, Annonaceae, Aquifoliaceae, Araliaceae, Bixaceae, Calophyllaceae, Caryocaraceae, Connaraceae, Ebenaceae, Lacistemataceae, Loganiaceae, Myristicaceae, Ochnaceae, Peraceae, Proteaceae, Salicaceae, Siparunaceae and Thymelaeaceae were represented by only one species each (Table 1).

Between May 2019 and July 2021, 24 fieldwork expeditions were undertaken. The interval between the expeditions varied due to rainy and dry periods which affected finding the same fertile species and lockdown because of the Covid-19 pandemic. During this time, 364 individuals distributed in 116 species, 82 genera and 39 families of angiosperms were collected. In October and November of 2023, two new collections were made, totalling 366 individuals, 118 species, 84 genera and 39 families (Table 1). A complete list of the vouchers is presented in Supplementary Material (Suppl. material 1).

**Figure 4.**
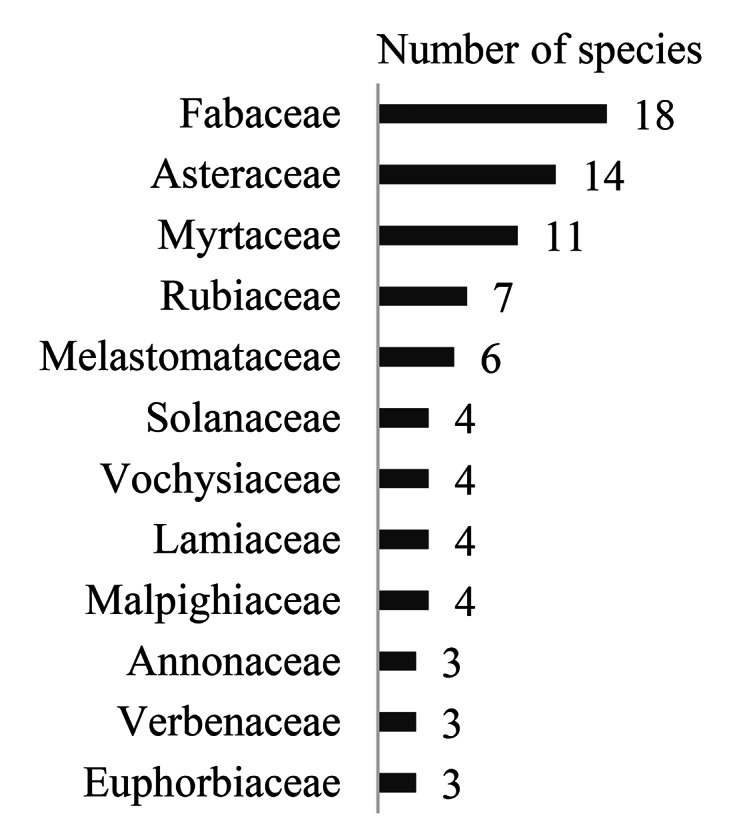
Field floristic surveys conducted in 2019, 2020, 2021 and 2023: families with the largest diversity in species.

Fabaceae (66), Asteraceae (22), Myrtaceae (26), Rubiaceae (23) and Melastomataceae (33) also had the greatest species diversity (Fig. 4). Aquifoliaceae, Araliaceae, Arecaceae, Caryocaraceae, Celastraceae, Connaraceae, Lacistemataceae, Loganiaceae, Moraceae, Myristicaceae, Ochnaceae, Peraceae, Polygalaceae, Proteaceae, Salicaceae, Siparunaceae, Styracaceae and Symplocaceae had only one taxon each (Table 1).

**Figure 5.**
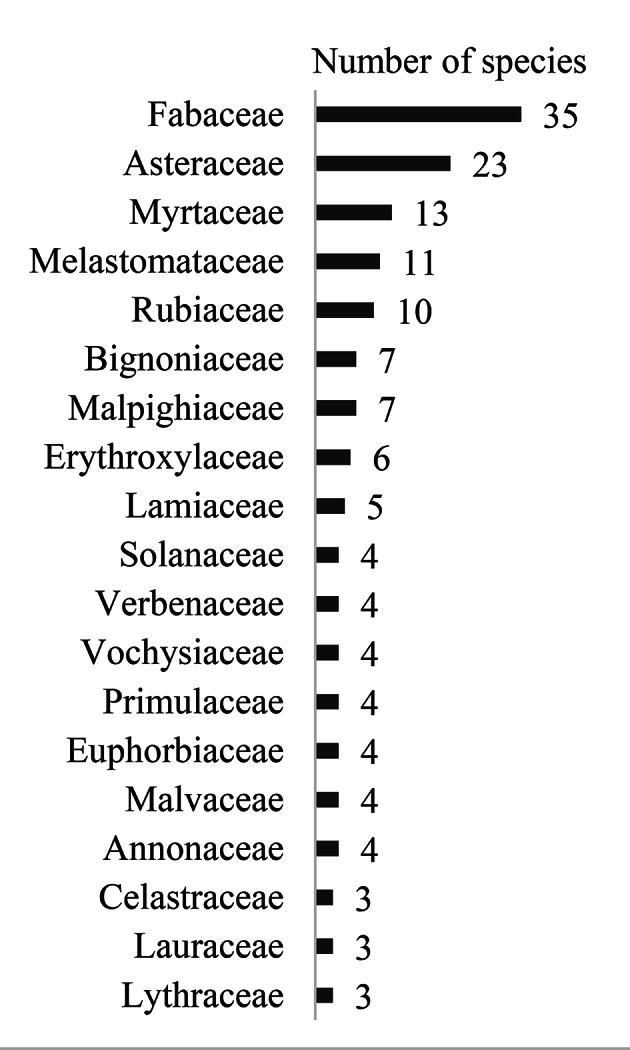
HRCB collection and field floristic survey: the families with the largest diversity in species.

For the HRCB and floristic surveys, there was a total of 192 species, 126 genera and 47 families (Table 1). However, 193 species were considered for the Reserve in Corumbataí, since *Syagrusromanzoffiana* (Cham.) Glassman occurs in this area, but was not collected. Popularly known as *jerivá*, this is a common species in the fragment and has been cited for the area (Pinheiro et al. 2021). The families with the greatest species diversity can be seen in Fig. 5. Exotic specimens of *Syzygiumjambos* (L.) Alston (*jambo*) and *Mangiferaindica* L. (*manga*) were collected, but are not on the list of taxa in the present study. Sterile individuals of *Pouteria* sp. and *Aspidosperma* sp. were also observed.

## Checklists

### Checklist of taxa from the Prof. Dr. Karl Arens Reserve

#### Liliopsida and Magnoliopsida

866BC759-A559-5942-AEBB-4C2C995CDADF

##### Notes

The list of taxa recorded in this study is presented in Table 1. Details of the examined material from the Dr. Karl Arens Reserve, Corumbataí (SP), are provided in Supplementary Material (Suppl. material 1).

## Discussion

Species such as *Xylopiaaromatica* (Lam.) Mart. (HRCB 17668), *Scheffleravinosa* (Cham. & Schltdl.) Frodin & Fiaschi (HRCB 32188), *Caryocarbrasiliense* Cambess. (HRCB 32249), *Sapiumglandulosum* (L.) Morong (HRCB 55742), *Bauhiniabrevipes* Vogel (HRCB 5516), *Leptolobiumdasycarpum* Vogel (HRCB 27646) and *Machaeriumacutifolium* Vogel (HRCB 17667) have been cited for the Reserve (Camargo and Arens 1967, Piccolo et al. 1971, Cesar et al. 1988, Pagano et al. 1989, Andena et al. 2005, Pinheiro 2013, Pinheiro et al. 2010, Pinheiro et al. 2021). However, those authors did not present their respective vouchers, even though there is herbarium material of them existing at HRCB and SPFR. Furthermore, no vouchers are cited for any other taxa in these works.

*Annonacrassiflora* Mart., *Syagrusromanzoffiana*, *Protiumheptaphyllum* (Aubl.) Marchand, *Monteverdiagonoclada* (Mart.) Biral, *Terminalia glabrescens* Mart., *Nectandramegapotamica* (Spreng.) Mez, *Ocoteaacutifolia* (Nees) Mez, *Trichiliahirta* L., *Campomanesiaguaviroba* (DC.) Kiaersk., *Saviadictyocarpa* Müll.Arg., *Esenbeckiagrandiflora* Mart., *Zanthoxylumrhoifolium* Lam. and *Copaiferalangsdorffii* Desf. are cited as present in the Reserve (Pinheiro et al. 2021); however, there is no testimony material in HRCB; however, with the exception of *Syagrus* and *Copaifera*, none of the cited species was found in our floristic survey. This may indicate either a change in the flora of the Reserve or incorrect identification. For example, the cited *Ocoteaacutifolia* is likely HRCB 5448, later recognised as *Ocoteacorymbosa* (Meisn.) Mez.

*Annonacacans* Warm., *Crotonfloribundus* Spreng. and *Piptocarphaaxillaris* (Less.) Baker are other species cited for the Reserve that have no testimony material and, according to Flora e Funga do Brasil (2024), occur in ombrophilous forests. On the other hand, *Perseawilldenovii* Kosterm. (HRCB 59275) was collected in 2012, but is not on any list in the works published. According to Moraes (personal observation), the only known individual in the Reserve died in 2013. *Ocoteacorymbosa* (Meisn.) Mez (HRCB 5448) was collected in 1984 and is also not mentioned in the recent studies.

Some exsiccatae in the collection have inconsistent localities, with two municipalities (Corumbataí and Itirapina), for example, HRCB 1490, HRCB 1325, HRCB 1328, HRCB 36700, HRCB 1310, HRCB 1591, HRCB 1592, HRCB 1388, HRCB 1465, HRCB 1477, HRCB 1497, HRCB 1598, HRCB 1102, HRCB 1103, HRCB 1104, HRCB 1105, HRCB 1106, HRCB 1121, HRCB 1122 and HRCB 1123). *Anacardiumhumile* A.St.-Hil., *Annonacornifolia* A.St.-Hil, and *Duguetiafurfuracea* (A.St.-Hil.) Saff. are amongst these vouchers and are species cited in a study only made with herbarium material (Pinheiro 2013). The specimens with a conflicting locality on their labels are not on the species list of the present study.

Pinheiro et al. (2021) determined 510 individuals from the Reserve, which were distributed in 103 species and 43 families. However, we only found two collections in HRCB made by the first author (HRCB 45190, *Peraglabrata* (Schott) Poepp. ex Baill. and HRCB 45191, *Daphnopsisfasciculata* (Meisn.) Nevling), both from 2003 and without a collection number (Table 1). Providing a voucher from analysed specimens is important for increasing the credibility of studies and enabling the location of the examined material. According to Baker et al. (2017), a reliable identification is linked to a well-prepared and cited voucher. Adequate documentation is fundamental to allow comparisons, improvements (Schilthuizen et al. 2015) or corrections. This practice forms the basis of collections and scientific reproducibility (Funk et al. 2005); however, the studies with the specimens from the Reserve have been neglected over the years.

**Figure 6.**
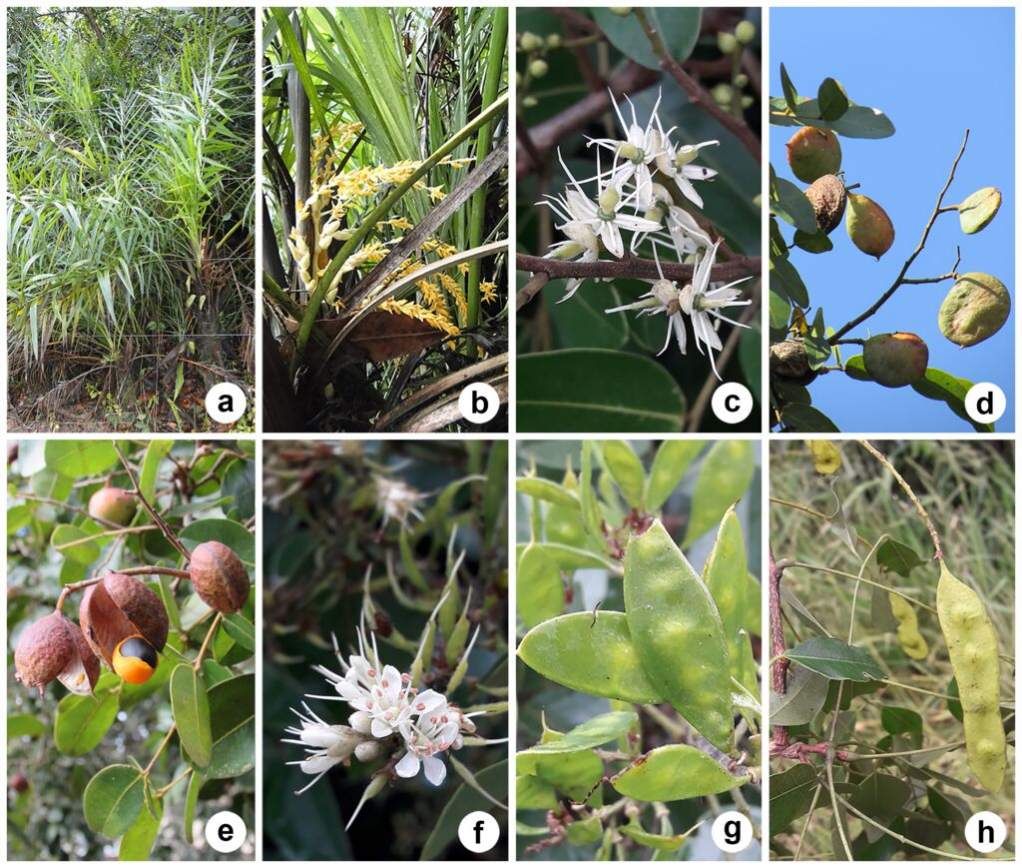
Some of the species mentioned in previous studies that did not have testimonial material deposited in the HRCB: **(a–b)** *Syagrusflexuosa*; **(c–e)** *Copaiferalangsdorffii*; **(f–h)** *Leptolobiumelegans*. Photos: Lilian S. Santos (a–f).

*Copaiferalangsdorffii*, *Syagrusflexuosa* (Mart.) Becc., *Leptolobiumelegans* Vogel, Eugeniacf.hiemalis Cambess. and *Myrcianeoclusiifolia* A.R.Lourenço & E.Lucas (Fig. 6) were added to the HRCB collection for the first time, although they were already cited for the Reserve. According to Pinheiro et al. (2021), *Copaiferalangsdorffii* is one of the most abundant species in the Reserve, as observed by the authors in the present study, particularly along the northern border of the fragment.

**Figure 7.**
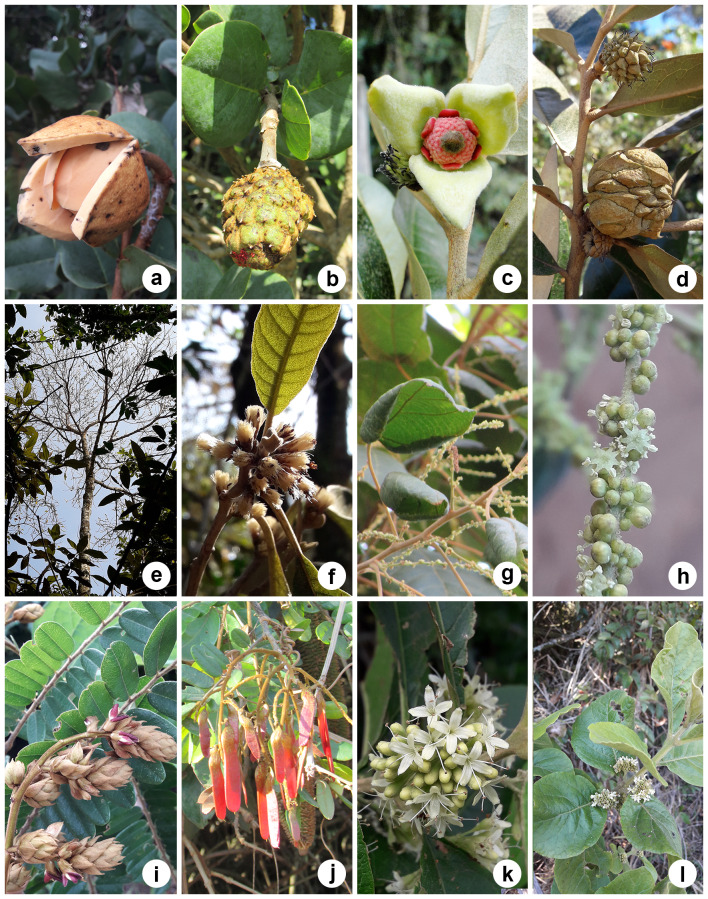
Some of the species not mentioned in previous studies and without testimonial material deposited in the HRCB: **(a-b)** *Annonacoriacea*; **(c–d)** *Duguetiafurfuracea*; **(e–f)** *Piptocarphamacropoda*; **(g–h)** *Alchorneaglandulosa*; **(i–j)** *Machaeriumnyctitans*; **(k–l)** *Aegiphilaverticillata*. Photos: Lilian S. Santos (a,b,e–l); Alessandra I. Coan (c–d).

On the other hand, *Annonacoriacea* Mart., *Duguetiafurfuracea, Guatteriaaustralis* A.St.-Hil., *Moquiniastrumpolymorphum* (Less.) G.Sancho, *Piptocarphamacropoda* (DC.) Baker, *Alchorneaglandulosa* Poepp. & Endl., *Machaeriumnyctitans* (Vell.) Benth., *Aegiphilaintegrifolia* (Jacq.) Moldenke, *Aegiphilaverticillata* Vell., *Cedrelafissilis* Vell., *Ficusguaranitica* Chodat, *Lueheagrandiflora* Mart. and *Guareaguidonia* (L.) Sleumer did not have testimony material, were not on species lists for the Reserve (Pinheiro et al. 2021) and were added to HRCB for the first time (Fig. 7). Despite *A.coriacea* being added to the HRCB collection for the first time, there are records of this species from Corumbataí in other herbaria (ESA 023328 and PEL 20336).

The floristic survey recorded fewer species and families than what was found in the herbarium. This could be related to the phenology of the plants and their fertile periods (Baker et al. 2017), as well as to the chance of finding plants with flowers or fruits (e.g. *Pouteria* sp. that was only observed in a vegetative state). The field of vision of the collector is impaired in denser vegetation and this may have influenced the lower number of taxa collected. The lower number of species may also be related to the difficulty some plants have in competing for light, since the *Cerradão* grows over the *Cerrado* *sensu stricto.* Although most of the collected specimens were found on the border and not in the middle of the fragment, the denser vegetation still harbours species that are not found elsewhere within the Reserve, such as *Andiravermifuga* (Mart.) Benth. and *Piptocarphamacropoda* (DC.) Baker. Despite being modest, differences were observed between the species diversity of the floristic survey and the HRCB collection. The family Annonaceae, for example, was represented by only one species in the herbarium and is now represented by four taxa (Table 1).

According to Durigan and Ratter (2006), the change in the physiognomy is accompanied by changes in the floristic composition. The densification of the vegetation makes it difficult for sunlight to enter, which affects the species from *cerrado* *sensu stricto* in the understorey (Pinheiro and Durigan 2012, Pinheiro et al. 2021). In the most closed areas in the middle of the fragment, dead individuals were observed with very suberised trunks. The absence of fires can be associated with a decrease in species in more open areas and an increase in large generalist species (Pinheiro et al. 2010, Pinheiro et al. 2021). Additionally, the absence of disturbances, such as fire and deforestation, contribute to an increase in tree cover to the detriment of grasses (Scholes and Archer 1997) and other plants more dependent on the sun. The single or few exsiccatae of *Aspidospermatomentosum* Mart., *Cochlospermumregium* (Mart. ex Schrank) Pilg., *Kielmeyeracoriacea* Mart. & Zucc., *Couepiagrandiflora* (Mart. & Zucc.) Benth., *Leptobalanushumilis* (Cham. & Schltdl.) Sothers & Prance and *Diospyroslasiocalyx* (Mart.) B.Walln., encountered in the HRCB collection, are old specimens that were not collected in the floristic inventory.

**Figure 8.**
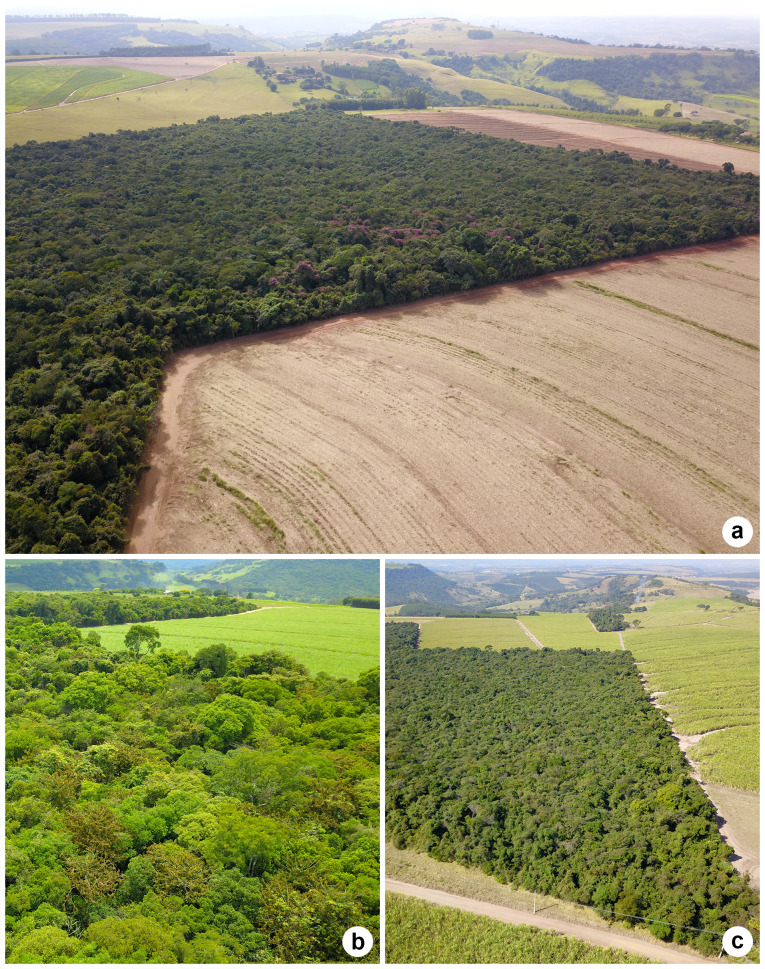
Aerial images from the Prof. Dr. Karl Arens Reserve: **(a)** May 2021; **(b)** December 2019; **(c)** July 2019. Photos: Bruno D. Borges (a–c).

**Figure 9.**
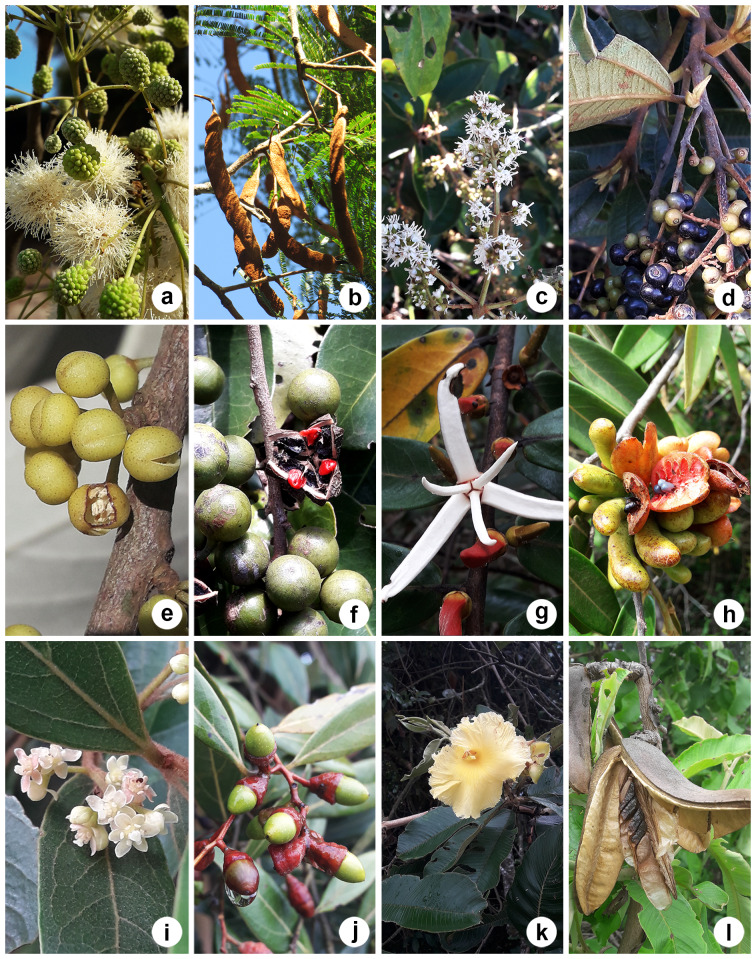
Some of the more common species from the Prof. Dr. Karl Arens Reserve: **(a–b)** *Anadenantheraperegrina*; **(c–d)** *Miconiarubiginosa*; **(e–f)** *Peraglabrata*; **(g–h)** *Xylopiaaromatica*; **(i–j)** *Virolasebifera*; **(k–l)** *Ocoteapulchella*; **(m–n)** *Amaiouaintermedia*; **(o–p)** *Qualeagrandiflora*. Photos: Lilian S. Santos (a–p).

Images taken by drones (Fig. 8) allowed the collector on the ground to have another angle of observation. However, during the period when the photographs were taken, only a predominance of vines in the families Malpighiaceae and Bignoniaceae, as well as crowns without apparent flowers or fruits, were observed. Most of the collections were made on the edge of the Reserve, where there is more sunlight. Many shrubs and treelets are notable on the edge, as well as trees of *Anadenantheraperegrina* (L.) Speg. and *Copaiferalangsdorffii* (Fig. 9), which are generalists and common in the Reserve (Pinheiro et al. 2021). In the interior of the fragment, many individuals also found along the border were observed, for example, *Peraglabrata, Ocoteapulchella* (Nees & Mart.) Mez, *Amaiouaintermedia* Mart. and *Virolasebifera* Aubl.

This study highlights the relevance of floristic surveys in the field and of herbarium material as sources of biological information, as well as the importance of documenting and publishing analysed vouchers. Although previous studies have been conducted in the Reserve, it was possible to collect species that have not been listed or lack testimony material, which reinforces the importance of floristic studies in already known areas. The influence of the lack of disturbances, such as fire, is noticeable, based on the change in the physiognomy and flora over the years.

In addition to the floristic diversity, the Reserve has a fauna comprised of various birds, for example, *Cariamacristata* (seriema), *Mimussaturninus* (sabiá-do-campo), *Tarabamajor* (choró-boi) and *Caracaraplancus* (carcará), as well as reptiles, arthropods, cervids and other small mammals (Santos, personal observation). The Prof. Dr. Karl Arens Reserve maintains its importance as an area used for research and to preserve species and it is one of the few *Cerrado* remnants in the State of São Paulo. An updated floristic list, now with the respective vouchers, is also relevant regarding academic training because there are plant biology field classes taught to undergraduates in the Reserve.

## Supplementary Material

XML Treatment for Liliopsida and Magnoliopsida

8A285F57-0FD9-5754-A9BE-68ADA0DD0DC6 10.3897/BDJ.13.e142217.suppl1 Supplementary material 1 List of the examined vouchers from Dr. Karl Arens Reserve, Corumbataí (SP) Data type Taxa Brief description List of the examined vouchers from Dr. Karl Arens Reserve, Corumbataí (SP), deposited in the Herbário Rioclarense (HRCB) of the Universidade Estadual Paulista (UNESP), Câmpus de Rio Claro. File: oo_1134995.pdf https://binary.pensoft.net/file/1134995 Lilian Silva Santos & Pedro Luís Rodrigues de Moraes

## Figures and Tables

**Table 1.** Checklist of the taxa from the Prof. Dr. Karl Arens Reserve; specimens obtained from the HRCB collection and field floristic survey in 2019, 2020, 2021 and 2023. Number of vouchers in brackets. Taxa organised, based on APG IV.

**Checklist of the taxa from the Prof. Dr. Karl Arens Reserve**		
**PHYLUM > CLASS > ORDER > Family > Species**	**HRCB**	**SURVEY**
**TRACHEOPHYTA**		
**MAGNOLIOPSIDA**		
**LAURALES**		
**Lauraceae**		
*Ocoteacorymbosa* (Meisn.) Mez	(1)	(6)
*Ocoteapulchella* (Nees & Mart.) Mez	(8)	(8)
*Perseawilldenovii* Kosterm.	(3)	
**Siparunaceae**		
*Siparunaguianensis* Aubl.	(4)	(3)
**MAGNOLIALES**		
**Annonaceae**		
*Annonacoriacea* Mart.		(7)
*Duguetiafurfuracea* (A.St.-Hil.) Saff.		(3)
*Guatteriaaustralis* A.St.-Hil.		(4)
*Xylopiaaromatica* (Lam.) Mart.	(3)	(4)
**Myristicaceae**		
*Virolasebifera* Aubl.	(8)	(5)
**LILIOPSIDA**		
**ARECALES**		
**Arecaceae**		
*Syagrusflexuosa* (Mart.) Becc.		(1)
**MAGNOLIOPSIDA**		
**PROTEALES**		
**Proteaceae**		
*Roupalamontana* Aubl.	(4)	(5)
**DILLENIALES**		
**Dilleniaceae**		
*Davillaelliptica* A.St.-Hil.	(1)	(1)
*Davillarugosa* Poir.	(4)	(2)
**CELASTRALES**		
**Celastraceae**		
*Monteverdiaevonymoides* (Reissek) Biral	(1)	
*Peritassacampestres* (Cambess.) A.C.Sm.	(5)	
*Plenckiapopulnea* Reissek	(1)	(3)
**OXALIDALES**		
**Connaraceae**		
*Connarussuberosus* Planch.	(4)	(1)
**MALPIGHIALES**		
**Calophyllaceae**		
*Kielmeyeracoriacea* Mart. & Zucc.	(2)	
**Caryocaraceae**		
*Caryocarbrasiliense* Cambess.	(8)	(5)
**Chrysobalanaceae**		
*Couepiagrandiflora* (Mart. & Zucc.) Benth.	(3)	
*Leptobalanushumilis* (Cham. & Schltdl.) Sothers & Prance	(1)	
**Erythroxylaceae**		
*Erythroxylumcampestre* A.St.-Hil.	(2)	
*Erythroxylumcuneifolium* (Mart.) O.E.Schulz	(4)	(4)
*Erythroxylumdaphnites* Mart.	(2)	
*Erythroxylumdeciduum* A.St.-Hil.	(1)	
*Erythroxylumpelleterianum* A.St.-Hil.	(3)	(1)
*Erythroxylumsuberosum* A.St.-Hil.	(5)	
**Euphorbiaceae**		
*Alchorneaglandulosa* Poepp. & Endl.		(1)
*Crotongnaphaloides* Schrad.	(4)	(2)
*Microstachysserrulata* (Mart.) Müll.Arg.	(3)	
*Sapiumglandulosum* (L.) Morong	(5)	(1)
**Lacistemataceae**		
*Lacistemahasslerianum* Chodat	(7)	(4)
**Malpighiaceae**		
*Banisteriopsiscampestris* (A.Juss.) Little	(1)	
*Banisteriopsisstellaris* (Griseb.) B.Gates		(4)
*Byrsonimacoccolobifolia* Kunth	(4)	(1)
*Byrsonimaintermedia* A.Juss.	(8)	(8)
*Byrsonimaverbascifolia* (L.) DC.	(4)	
*Heteropterysumbellata* A.Juss.	(3)	
*Peixotoatomentosa* A.Juss.	(1)	(1)
**Ochnaceae**		
*Ourateaspectabilis* (Mart.) Engl.	(3)	(4)
**Peraceae**		
*Peraglabrata* (Schott) Baill.	(14)	(10)
**Salicaceae**		
*Caseariasylvestris* Sw.	(7)	(5)
**FABALES**		
**Fabaceae**		
*Anadenantheraperegrina* (L.) Speg.	(4)	(5)
*Andirahumilis* Benth.	(2)	
*Andiravermifuga* (Mart.) Benth.	(4)	(1)
*Bauhiniabrevipes* Vogel	(3)	(4)
*Bauhiniaholophylla* (Bong.) Steud.	(3)	(2)
Cerradicolacf.decumbens (Benth.) L.P.Queiroz	(3)	
*Chamaecristacathartica* (Mart.) H.S.Irwin & Barneby	(4)	
*Chamaecristadesvauxii* (Collad.) Killip	(3)	
*Chamaecristaflexuosa* (L.) Greene	(6)	(3)
*Chamaecristarotundifolia* (Pers.) Greene	(3)	
*Clitoriadensiflora* (Benth.) Benth.	(2)	
*Copaiferalangsdorffii* Desf.		(6)
*Crotalariamaypurensis* Kunth	(2)	
*Crotalariamicans* Link		(2)
CrotalariapallidaAitonvar.obovata (G.Don) Polhill	(1)	
*Dahlstedtiamuehlbergiana* (Hassl.) M.J.Silva & A.M.G.Azevedo	(1)	
Dalbergiafrutescens(Vell.)Brittonvar.frutescens	(1)	
*Dalbergiamiscolobium* Benth.	(1)	(3)
*Desmodiumtortuosum* (Sw.) DC.	(1)	
*Dimorphandramollis* Benth.	(2)	(4)
*Indigoferahirsuta* L.	(1)	
*Leptolobiumdasycarpum* Vogel	(4)	(4)
*Leptolobiumelegans* Vogel		(8)
*Machaeriumacutifolium* Vogel	(3)	(4)
*Machaeriumnyctitans* (Vell.) Benth.		(3)
*Machaeriumvillosum* Vogel	(1)	
*Mimosadebilis* Willd.	(2)	(1)
MimosaxanthocentraMart.var.subsericea (Benth.) Barneby	(3)	(1)
*Senegalialowei* (L.Rico) Seigler & Ebinger	(2)	
*Sennapendula* (Willd.) H.S.Irwin & Barneby	(1)	(2)
*Sennarugosa* (G.Don) H.S.Irwin & Barneby	(4)	(5)
*Stryphnodendronadstringens* (Mart.) Coville	(5)	
*Stryphnodendronrotundifolium* Mart.	(4)	(8)
*Stylosanthesscabra* Vogel	(1)	
*Stylosanthesviscosa* (L.) Sw.	(1)	
**Polygalaceae**		
*Bredemeyerafloribunda* Willd.	(2)	(3)
*Polygalapoaya* Mart.	(6)	
**ROSALES**		
**Moraceae**		
*Ficusguaranitica* Chodat		(3)
**MYRTALES**		
**Lythraceae**		
*Cupheathymoides* Cham. & Schltdl.	(1)	
*Diplusodonvirgatus* Pohl	(6)	(4)
*Lafoensiapacari* A.St.-Hil.	(1)	(3)
**Melastomataceae**		
*Leandraaurea* (Cham.) Cogn.	(2)	
*Miconiaalbicans* (Sw.) Triana	(5)	(1)
*Miconiafallax* DC.	(2)	
*Miconiaflammea* Casar.	(3)	(3)
*Miconialigustroides* (DC.) Naudin	(9)	(9)
*Miconiapaucidens* DC.	(1)	
*Miconiarubiginosa* (Bonpl.) DC.	(9)	(14)
*Miconiasellowiana* Naudin	(2)	(4)
*Miconiastenostachya* DC.	(4)	(2)
*Microliciapolystemma* Naudin	(1)	
*Pleromastenocarpum* (DC.) Triana	(4)	
**Myrtaceae**		
*Blepharocalyxsalicifolius* (Kunth) O.Berg	(2)	(1)
*Campomanesiapubescens* (DC.) O.Berg	(6)	(2)
*Eugeniaaurata* O.Berg	(2)	(1)
*Eugeniabimarginata* DC.	(4)	(3)
Eugeniacf.hiemalis Cambess.		(3)
*Eugeniapunicifolia* (Kunth) DC.	(4)	(5)
*Myrciabela* Cambess.	(5)	(1)
*Myrciaguianensis* (Aubl.) DC.	(4)	(7)
*Myrcianeoclusiifolia* A.R.Lourenço & E.Lucas		(1)
*Myrciasplendens* (Sw.) DC.	(2)	(1)
*Myrciatomentosa* (Aubl.) DC.	(2)	
*Myrciariafloribunda* (Willd.) O.Berg	(3)	(1)
*Psidiumgrandifolium* DC.	(2)	
**Vochysiaceae**		
*Qualeacordata* Spreng.	(1)	(2)
*Qualeagrandiflora* Mart.	(3)	(5)
*Qualeamultiflora* Mart.	(3)	(5)
*Vochysiatucanorum* Mart.	(10)	(6)
**SAPINDALES**		
**Meliaceae**		
*Cedrelafissilis* Vell.		(3)
*Guareaguidonia* (L.) Sleumer		(1)
**Sapindaceae**		
*Serjaniaerecta* Radlk.	(2)	
*Talisiaangustifolia* Radlk.	(1)	
**MALVALES**		
**Bixaceae**		
*Cochlospermumregium* (Schrank) Pilg.	(1)	
**Malvaceae**		
*Byttneriasagittifolia* A.St.-Hil.	(1)	
*Eriothecagracilipes* (K.Schum.) A.Robyns	(2)	(3)
*Lueheagrandiflora* Mart.		(1)
*Pavoniamalacophylla* (Link & Otto) Garcke	(1)	
**Thymelaeaceae**		
*Daphnopsisfasciculata* (Meisn.) Nevling	(5)	
**CARYOPHYLLALES**		
**Nyctaginaceae**		
*Guapiranoxia* (Netto) Lundell	(8)	(3)
*Neeatheifera* Oerst.	(5)	(3)
**ERICALES**		
**Ebenaceae**		
*Diospyroslasiocalyx* (Mart.) B.Walln.	(1)	
**Primulaceae**		
Myrsinecf.coriacea (Sw.) Roem. & Schult.	(1)	
*Myrsineguianensis* (Aubl.) Kuntze	(4)	
*Myrsinelancifolia* Mart.	(1)	(1)
*Myrsineumbellata* Mart.	(4)	(2)
**Sapotaceae**		
*Pouteriaramiflora* (Mart.) Radlk.	(1)	
*Pouteriatorta* (Mart.) Radlk.	(3)	
**Styracaceae**		
*Styraxcamporum* Pohl	(7)	(6)
*Styraxferrugineus* Nees & Mart.	(4)	
**Symplocaceae**		
*Symplocosoblongifolia* Casar.	(3)	
*Symplocospubescens* Klotzsch ex Benth.	(4)	(1)
**SOLANALES**		
**Solanaceae**		
*Cestrummariquitense* Kunth	(3)	(2)
Solanumcf.didymum Dunal		(1)
*Solanumgranulosoleprosum* Dunal	(1)	(3)
*Solanumlycocarpum* A.St.-Hil.	(5)	(4)
**GENTIANALES**		
**Apocynaceae**		
*Aspidospermatomentosum* Mart.	(1)	
*Tabernaemontanacatharinensis* A.DC.	(1)	
**Loganiaceae**		
*Strychnosbicolor* Progel	(3)	(1)
**Rubiaceae**		
*Amaiouaintermedia* Mart.	(8)	(7)
*Cordieraobtusa* (K.Schum.) Kuntze	(2)	
*Cordierasessilis* (Vell.) Kuntze		(2)
*Declieuxiafruticosa* (Roem. & Schult.) Kuntze	(1)	
*Palicoureahoffmannseggiana* (Schult.) Borhidi	(2)	(1)
*Palicoureamarcgravii* A.St.-Hil.	(3)	(4)
*Palicourearigida* Kunth	(6)	
*Palicoureasessilis* (Vell.) C.M.Taylor	(5)	(3)
*Palicoureaviolacea* (Aubl.) A.Rich	(5)	(4)
*Tocoyenaformosa* (Cham. & Schltdl.) K.Schum.	(4)	(2)
**LAMIALES**		
**Bignoniaceae**		
*Adenocalymmaperegrinum* (Miers) L.G.Lohmann		(2)
*Anemopaegmaacutifolium* DC.	(1)	
*Anemopaegmaarvense* (Vell.) J.F.Souza	(1)	
Handroanthuscf.ochraceus (Cham.) Mattos	(1)	
*Jacarandacaroba* (Vell.) DC.	(3)	(2)
*Jacarandadecurrens* Cham.	(1)	
*Zeyheriamontana* Mart.	(1)	
**Lamiaceae**		
*Aegiphilaintegrifolia* (Jacq.) Moldenke		(2)
*Aegiphilaverticillata* Vell.		(1)
*Cyanocephaluslippioides* (Benth.) Harley & J.F.B.Pastore	(5)	
*Medusanthaeriophylla* (Benth.) Harley & J.F.B.Pastore	(3)	(1)
*Medusantha* sp.		(2)
**Verbenaceae**		
*Lantanacamara* L.		(2)
*Lantanafucata* Lindl.	(1)	(1)
*Lippiaoriganoides* Kunth	(2)	(3)
*Stachytarphetacayennensis* (Rich.) Vahl	(1)	
**AQUIFOLIALES**		
**Aquifoliaceae**		
*Ilexcerasifolia* Reissek	(3)	(4)
**ASTERALES**		
**Asteraceae**		
Achyroclinecf.satureioides (Lam.) DC.	(2)	(1)
*Aldamaarenaria* (Baker) E.E.Schill. & Panero	(4)	
*Baccharisdracunculifolia* DC.	(4)	(1)
*Chrestasphaerocephala* DC.	(3)	
*Chromolaenaodorata* (L.) R.M.King & H.Rob.		(1)
*Chromolaenasqualida* (DC.) R.M.King & H.Rob.	(2)	(1)
*Chrysolaenacognata* (Less.) Dematt.	(2)	
*Grazieliadimorpholepis* (Baker) R.M.King & H.Rob.	(1)	
*Heterocondylusalatus* (Vell.) R.M.King & H.Rob.	(5)	
*Hoehnephytumtrixoides* (Gardner) Cabrera	(1)	
*Lepidaploa* sp.1		(2)
*Lepidaploa* sp.2		(1)
*Lessingianthusbardanoides* (Less.) H.Rob.	(1)	
*Moquiniastrumbarrosoae* (Cabrera) G.Sancho	(3)	(3)
*Moquiniastrumpolymorphum* (Less.) G.Sancho		(4)
*Moquiniastrumpulchrum* (Cabrera) G.Sancho	(4)	(1)
*Piptocarphamacropoda* (DC.) Baker		(1)
*Piptocarpharotundifolia* (Less.) Baker	(8)	
*Pterocaulonlanatum* Kuntze		(2)
Trixiscf.antimenorrhoea (Schrank) Kuntze		(1)
*Vernonanthuraferruginea* (Less.) H.Rob.	(1)	
*Vernonanthurapolyanthes* (Spreng.) Vega & Dematteis		(1)
Vernonanthuracf.rubriramea (DC.) Loeuille & P.N.Soares		(2)
**APIALES**		
**Araliaceae**		
*Scheffleravinosa* (Cham. & Schltdl.) Frodin & Fiaschi	(6)	(5)
