# Supplementary material for: Woody flora of the Prof. Dr. Karl Arens Reserve, Corumbataí, São Paulo, Brazil
Source: Biodivers Data J. 2025 Feb 3;13:e142217. doi: 10.3897/BDJ.13.e142217 (PMC11811714; doi:10.3897/BDJ.13.e142217)
Supplement: Supplementary material 1 — List of the examined vouchers from Dr. Karl Arens Reserve, Corumbataí (SP) [file bdj-13-e142217-s001.pdf]

Supplementary Material to "Woody flora of the Prof. Dr. Karl Arens Reserve, Corumbataí (SP)"

List of the examined vouchers from Dr. Karl Arens Reserve, Corumbataí (SP), deposited in the Herbário Rioclarense (HRCB) of the Universidade Estadual Paulista (UNESP), Câmpus de Rio Claro.

Annonaceae

Annona coriacea Mart.: BRASIL. SÃO PAULO: Corumbataí, Reserva de Cerrado da UNESP, 13.VI.2019, fr., *L.S. Santos et al. 78* (HRCB); idem, 13.VII.2019, fr., *L.S. Santos et al.* 117 (HRCB); idem, 10.I.2020, fl., *L.S. Santos & R.N. Leite 248* (HRCB); idem, 29.X.2020, bud, *L.S. Santos et al. 282* (HRCB); idem, 26.I.2021, fl., *L.S. Santos & L.P. Elias 307* (HRCB); idem, 16.VI.2021, fr., *L.S. Santos et al. 372* (HRCB); idem, 4.XII.2019, fl., *P.L.R. Moraes et al.* 5525 (HRCB).

Duguetia furfuracea (A.St.-Hil.) Saff.: BRASIL. SÃO PAULO: Corumbataí, Reserva de Cerrado da UNESP, 18.V.2019, fl. & fr., *L.S. Santos et al. 61* (HRCB); idem, 1.VIII.2019, fr., *L.S. Santos et al. 155* (HRCB); idem, 29.X.2020, fr., *L.S. Santos et al. 283* (HRCB).

Guatteria australis A.St.-Hil.: BRASIL. SÃO PAULO: Corumbataí, Reserva de Cerrado da UNESP, 18.V.2019, fr., *L.S. Santos et al. 12* (HRCB); idem, 18.V.2019, *L.S. Santos et al. 46* (HRCB); idem, 4.XII.2019, fl. & fr., *L.S. Santos et al. 210* (HRCB); idem, 18.V.2021, fl., *L.S. Santos et al. 281* (HRCB).

Xylopia aromatica (Lam.) Mart.: BRASIL. SÃO PAULO: Corumbataí, Reserva de Cerrado, 6.VII.1982, bud, *L. Cordeiro* (HRCB 2684); idem, Cerrado de Corumbataí, 21.X.1983, bud, fl., *L. Cordeiro* (HRCB 3659); idem, 26.X.1994, bud, fr., *Adriane 1* (HRCB); idem, Reserva de Cerrado da UNESP, 18.V.2019, fr., *L.S. Santos et al. 51* (HRCB); idem, 13.VI.2019, fr., *L.S. Santos et al. 66* (HRCB); idem, 30.IX.2019, bud, *L.S. Santos & T.S. El Hindi 187* (HRCB); idem, 4.XII.2019, bud, fl., *L.S. Santos et al. 215* (HRCB).

Apocynaceae

Aspidosperma tomentosum Mart.: BRASIL. SÃO PAULO: Corumbataí, 2.IX.1989, bud, fl., *L.C. Saraiva, 78* (HRCB).

Tabernaemontana catharinensis A.DC.: BRASIL. SÃO PAULO: Corumbataí, 7.XI.1984, fl., *L. Cordeiro 14* (HRCB).

Aquifoliaceae

Ilex cerasifolia Reissek: BRASIL. SÃO PAULO: Corumbataí, Cerrado de Corumbataí, 30.VI.1981, fr., *O. Cesar & S.N. Pagano 35* (HRCB); idem, Cerrado de Corumbataí, 30.VI.1981, fr., *O. Cesar & S.N. Pagano 29* (HRCB); idem, 24.II.2000, fr., *C.E. Carneiro et al. 31* (HRCB); idem, Reserva de Cerrado da UNESP, 13.VII.2019, fl., *L.S. Santos et al. 120* (HRCB); idem, 11.XII.2019, fr., *L.S. Santos & L.P. Elias 222* (HRCB); idem, 4.XII.2019, fr., *P.L.R. Moraes et al. 5516* (HRCB); idem, 4.XII.2019, fr., *P.L.R. Moraes et al. 5533* (HRCB).

Araliaceae

Schefflera vinosa (Cham. & Schltdl.) Frodin & Fiaschi: BRASIL. SÃO PAULO: Corumbataí, Cerrado de Corumbataí, 19.XII.1977, bud, fl., *O. Aulino* (HRCB 1124); idem, Cerrado de Corumbataí, 30.VI.1981, bud, *O. Cesar & S.N. Pagano 25* (HRCB); idem, Cerrado de Corumbataí, 24.VIII.1982, bud, fl. & fr., *O. Cesar & S.N. Pagano 59* (HRCB); idem, Cerrado de Corumbataí, 31.VIII.1983, fr., *C.S. Barbieri et al. 1* (HRCB); idem, 24.II.2000, bud, fl., *V.B. Ziparro et al. 1898* (HRCB); idem, Reserva da UNESP, 10.VIII.2006, fl. & fr., *J.A. Lombardi et al. 6407* (HRCB); idem,Reserva de Cerrado da UNESP, 18.V.2019, fl. & fr., *L.S. Santos et al. 8* (HRCB); idem, 13.VII.2019, fr., *L.S. Santos et al. 107* (HRCB); idem, 26.I.2021, fr., *L.S. Santos & L.P. Elias 306* (HRCB); idem, 6.VII.2021, bud, fl., *L.S. Santos et al. 339* (HRCB); idem, 18.V.2021, bud, fl., *L.S. Santos et al. 361* (HRCB).

Arecaceae

Syagrus flexuosa (Mart.) Becc.: BRASIL. SÃO PAULO: Corumbataí, Reserva de Cerrado da UNESP, 18.V.2019, fl. & fr., *L.S. Santos et al. 13* (HRCB).

Asteraceae

Achyrocline cf. *saturejoides* (Lam.) DC.: BRASIL. SÃO PAULO: Corumbataí, Reserva de Cerrado, 25.IX.1962, *H. Amaral* (HRCB 1232); XII.1963, *J. Mattos* (HRCB 1240); Reserva de Cerrado da UNESP, 8.IV.2021, fl., *L.S. Santos et al. 333* (HRCB).

Aldama arenaria (Baker) E.E.Schill. & Panero: BRASIL. SÃO PAULO: Corumbataí, Cerrado de Corumbataí, 20.II.1978, fl., *J.C. Toledo 10* (HRCB); idem, Cerrado de Corumbataí, 22.II.1983, fl., *C.M. Beltrati 4* (HRCB); idem, 5.III.1985, fl., *L.C. Saraiva 44* (HRCB); idem, II.1996, fl., *V.T. Rampin 834* (HRCB).

Baccharis dracunculifolia DC.: BRASIL. SÃO PAULO: Corumbataí, Cerrado de Corumbataí, 7.XII.1982, fl., *M.J.O. Campos 58* (HRCB); idem, 25.I.1984, fl., J.*A. Mendes* (HRCB 3995); idem, 24.II.2000, fl., *C.E. Carneiro et al. 24* (HRCB); idem, 24.II.2000, fl., *V.B. Ziparro et al. 1891* (HRCB); idem, Reserva de Cerrado da UNESP, 12.III.2020, bud, fl., *L.S. Santos & G.G. Queiros 271* (HRCB).

Chresta sphaerocephala DC.: BRASIL. SÃO PAULO: Corumbataí, Cerrado de Corumbataí, 28.VI.1983, fl., *L.C. Saraiva 23* (HRCB); idem, Cerrado de Corumbataí, 31.VIII.1983, fl., *I.V. Pereira 8* (HRCB); idem, 11.VII.1984, fl., *C.M. Beltrati 69* (HRCB).

Chromolaena odorata (L.) R.M.King & H.Rob.: BRASIL. SÃO PAULO: Corumbataí, Reserva de Cerrado da UNESP, 12.III.2020, fl., *L.S. Santos & G.G. Queiros 272* (HRCB).

Chromolaena squalida (DC.) R.M.King & H.Rob.: BRASIL. SÃO PAULO: Corumbataí, Reserva de Cerrado, 18.V.1977, fl., *G. Marinis & O. Cesar 21* (HRCB); idem, Corumbataí, 15.VIII.1984, fr., *Turma Biologia* (HRCB 4661); Reserva de Cerrado da UNESP, 14.VIII.2019, fl. & fr., *L.S. Santos et al. 172* (HRCB).

Chrysolaena cognata (Less.) Dematt.: BRASIL. SÃO PAULO: Corumbataí, Reserva de Cerrado, 14.IV.1962, fl., *H. Vitti* (HRCB 1231); fl., *n.d.* (HRCB 1225).

Grazielia dimorpholepis (Baker) R.M.King & H.Rob.: BRASIL. SÃO PAULO: Corumbataí, 9.II.1984, fl., *O. Cesar 143* (HRCB).

Heterocondylus alatus (Vell.) R.M.King & H.Rob.: BRASIL. SÃO PAULO: Corumbataí, Cerrado de Corumbataí, 7.VII.1981, fl., *O. Cesar & S.N. Pagano 23* (HRCB); idem, Cerrado de Corumbataí, 31.VIII.1983, fr., *I.V. Pereira 6* (HRCB); idem, Cerrado de Corumbataí, 13.VII.1981, fl., *O. Cesar & S.N. Pagano 30* (HRCB); idem, 15.VIII.1984, fl. & fr., *Turma-Biologia* (HRCB 4667); idem, 21.VIII.1985, fl., *A. Feddersen Jr. 6* (HRCB).

Hoehnephytum trixoides (Gardner) Cabrera: BRASIL. SÃO PAULO: Corumbataí, 17.VIII.1982, bud, fl., *M.J.O. Campos 13* (HRCB).

Lepidaploa sp.1: BRASIL. SÃO PAULO: Corumbataí, Reserva de Cerrado da UNESP, 18.V.2019, fl., *L.S. Santos et al. 16* (HRCB); idem, 1.VIII.2019, fl., *L.S. Santos et al. 131* (HRCB).

Lepidaploa sp.2: BRASIL. SÃO PAULO: Corumbataí, Reserva de Cerrado da UNESP, bud, fl., *L.S. Santos et al. 143* (HRCB).

Lessingianthus bardanoides (Less.) H.Rob.: BRASIL. SÃO PAULO: Corumbataí, Cerrado de Corumbataí, 29.III.1983, fl., *M.J.O. Campos 91* (HRCB).

Moquiniastrum barrosoae (Cabrera) G.Sancho: BRASIL. SÃO PAULO: Corumbataí, Cerrado de Corumbataí, 30.VIII.1982, fl., *M.J.O. Campos 6* (HRCB); idem, Cerrado de Corumbataí, 15.VIII.1984, fl., *Turma Biologia* (HRCB 4665); Reserva da UNESP, 10.VIII.2006, fl., *J.A. Lombardi et al. 6410* (HRCB); idem, Reserva de Cerrado da UNESP, 1.VIII.2019, fl., *L.S. Santos et al. 122* (HRCB); idem, 14.VIII.2019, fl., *L.S. Santos et al. 171* (HRCB); idem, 6.VII.2021, fl., *L.S. Santos et al. 344* (HRCB).

Moquiniastrum polymorphum (Less.) G.Sancho: BRASIL. SÃO PAULO: Corumbataí, Reserva de Cerrado da UNESP, 4.XII.2019, fl., *P.L.R. Moraes et al.* 5524 (HRCB); idem, 11.XII.2019, fl., *L.S. Santos & L.P. Elias 191* (HRCB); idem, 26.I.2021, fl., *L.S. Santos & L.P. Elias 300* (HRCB); idem, 11.III.2021, fl., *L.S. Santos et al. 322* (HRCB).

Moquiniastrum pulchrum (Cabrera) G.Sancho: BRASIL. SÃO PAULO: Corumbataí, Cerrado de Corumbataí, 30.VI.1981, fl., *O. Cesar & S.N. Pagano 24* (HRCB); idem, 17.VIII.1982, fl., *M.J. Campos 8* (HRCB); idem, 31.VIII.1984, fr., fl., *S.N. Pagano 617* (HRCB); idem, Reserva da UNESP, 10.VIII.2006, fl., *J.A. Lombardi et al. 6409* (HRCB); idem,Reserva de Cerrado da UNESP, 13.VI.2019, fl., *L.S. Santos et al. 65* (HRCB).

Piptocarpha macropoda (DC.) Baker: BRASIL. SÃO PAULO: Corumbataí, Reserva de Cerrado da UNESP, 14.VIII.2019, fr., *L.S. Santos et al. 166* (HRCB).

Piptocarpha rotundifolia (Less.) Baker: BRASIL. SÃO PAULO: Corumbataí, Reserva de Cerrado, 12.XI.1962, fl. & fr., *H. Amaral* (HRCB 1229); Reserva de Cerrado, 5.VII.1963, bud, *C. Moura 49* (HRCB); idem, Reserva de Cerrado, 29.VIII.1966, fl. & fr., *H. Vitti* (HRCB 1228); Reserva de Cerrado de Corumbataí, 29.VIII.1966, fr., *H. Vitti* (HRCB 1230); 9.II.1984, fl., *O. Cesar 153* (HRCB); idem, Cerrado de Corumbataí, 13.VII.1981, fr., *O. Cesar & S.N. Pagano 36* (HRCB); idem, 21.II.1984, fl. & fr., *J.A. Mendes 8* (HRCB); idem, 31.VIII.1984, fl. & fr., *S.N. Pagano 606* (HRCB).

Pterocaulon lanatum Kuntze: BRASIL. SÃO PAULO: Corumbataí, Reserva de Cerrado, 8.IV.2021, fr., *L.S. Santos et al. 328* (HRCB); idem, 3.V.2021, fr., *L.S. Santos & L.P. Elias 342* (HRCB).

Trixis cf. *antimenorrhoea* (Schrank) Kuntze: BRASIL. SÃO PAULO: Corumbataí, Reserva de Cerrado da UNESP, 1.VIII.2019, fl., *L.S. Santos et al. 140* (HRCB).

Vernonanthura ferruginea (Less.) H.Rob.: BRASIL. SÃO PAULO: Corumbataí, 15.VIII.1984, fr., *Turma Biologia* (HRCB 4666).

Vernonanthura cf. *rubriramea* (Mart. ex DC.) Loeuille & P.N.Soares: BRASIL. SÃO PAULO: Corumbataí, Reserva de Cerrado da UNESP, 18.V.2019, fl., *L.S. Santos et al. 42* (HRCB); idem, 18.V.2021, fl., *L.S. Santos et al. 356* (HRCB).

Vernonanthura polyanthes (Sprengel) Vega & Dematteis: BRASIL. SÃO PAULO: Corumbataí, Reserva de Cerrado da UNESP, 1.VIII.2019, fl., *L.S. Santos et al. 135* (HRCB).

Bignoniaceae

Adenocalymma peregrinum (Miers) L.G.Lohmann: BRASIL. SÃO PAULO: Corumbataí, Reserva de Cerrado da UNESP, 30.IX.2019, bud, fl., *L.S. Santos & T.S. El Hindi 199* (HRCB); idem, 26.I.2021, bud, fl., *L.S. Santos & L.P. Elias 297* (HRCB).

Anemopaegma acutifolium DC.: BRASIL. SÃO PAULO: Corumbataí, 26.X.1994, bud, *M.A. de Assis 450* (HRCB).

Anemopaegma arvense (Vell.) Stellfeld ex J.F.Souza: BRASIL. SÃO PAULO: Corumbataí, 26.X.1994, fl., *M.A. de Assis 448* (HRCB).

Handroanthus cf. *ochraceus* (Cham.) Mattos: BRASIL. SÃO PAULO: Corumbataí, Cerrado de Corumbataí, 25.IX.1964, fl., *H. Vitti* (HRCB 1201).

Jacaranda caroba (Vell.) DC.: BRASIL. SÃO PAULO: Corumbataí, Cerrado de Corumbataí, 15.IX.1960, bud, fl., *n.d.* (HRCB 1145); Corumbataí, 11.IX.1979, bud, fl., *O. Cesar* (HRCB 1097); idem, Cerrado de Corumbataí, 31.VIII.1983, bud, fl., *I.V. Pereira 3* (HRCB); idem,Reserva de Cerrado da UNESP, 12.III.2020, fr., *L.S. Santos & G.G. Queiros 150* (HRCB); idem, 14.VIII.2019, bud, fl., *L.S. Santos et al. 158* (HRCB).

Jacaranda decurrens Cham.: BRASIL. SÃO PAULO: Corumbataí, 26.X.1994, bud, fl., *M.A. de Assis 449* (HRCB).

Zeyheria montana Mart.: BRASIL. SÃO PAULO: Corumbataí, Cerrado de Corumbataí, 17.IV.1965, bud, fr., *H. Vitti* (HRCB 1207).

Bixaceae

Cochlospermum regium (Mart. ex Schrank) Pilg.: BRASIL. SÃO PAULO: Corumbataí, 31.VIII.1984, fl., *S.N. Pagano 632* (HRCB).

Calophyllaceae

Kielmeyera coriacea Mart. & Zucc.: BRASIL. SÃO PAULO: Corumbataí, Reserva de Cerrado, 25.IV.1962, bud, *H. Amaral* (HRCB 1303); Reserva de Cerrado, bud, *H. Amaral* (HRCB 1304).

Caryocaraceae

Caryocar brasiliense Cambess.: BRASIL. SÃO PAULO: Corumbataí, Reserva de Cerrado, 29.III.1965, fl., *H. Vitti* (HRCB 1217); Reserva de Cerrado, 29.III.1976, fl., *G. Marinis e O. Cesar* (HRCB 1218); idem, 11.IX.1979, bud, *O. Cesar* (HRCB 1100); idem, Cerrado de Corumbataí, 31.VIII.1983, bud, fl., *I.V. Pereira 2* (HRCB); idem, 15.VIII.1984, fl., *Turma-Biologia* (HRCB 4656); 5.X.1996, bud, fl., *A.M.S. Pitolli 10* (HRCB); idem, Área de proteção ambiental sob responsabilidade da UNESP, 24.II.2000, bud, fl., *V.F.O. Miranda et al. 169* (HRCB); idem, 24.II.2000, fl., *C.E. Carneiro 23* (HRCB); idem, Reserva de Cerrado da UNESP, 30.IX.2019, fl., *L.S. Santos & T.S. El Hindi 182* (HRCB); idem, 17.XII.2019, fr., *L.S. Santos & L.P. Elias 240* (HRCB); idem, 10.I.2020, fr., *L.S. Santos & R.N. Leite 253* (HRCB); idem, 29.X.2020, bud, fl., *L.S. Santos et al. 284* (HRCB); idem, 26.I.2021, fr., *L.S. Santos & L.P. Elias 313* (HRCB).

Celastraceae

Monteverdia evonymoides (Reissek) Biral: BRASIL. SÃO PAULO: Corumbataí, 20.IX.1999, fl., *M. Cortezi* (HRCB 33076).

Peritassa campestris (Cambess.) A.C.Sm.: BRASIL. SÃO PAULO: Corumbataí, Reserva de Cerrado, 21.XII.1962, bud, fl., *J. Mattos* (HRCB 1308); idem, 10.VIII.1981, bud, fl., *O. Cesar & S.N. Pagano 37* (HRCB); idem, 30.VIII.1982, bud, fl., *M.J.O. Campos 24* (HRCB 6096); reserva da UNESP, 18.XI.2005, fr., *J.A. Lombardi 6210* (HRCB); idem, n.d., fl. & fr., *M.J.O. Campos 24* (HRCB 6473).

Plenckia populnea Reissek: BRASIL. SÃO PAULO: Corumbataí, 26.X.1994, bud, fl., *Adriana et al. 8* (HRCB); idem, Reserva de Cerrado da UNESP, 18.V.2019, fr., *L.S. Santos et al. 45* (HRCB); idem, 12.II.2020, fl., *L.S. Santos & M.M. Suemitsu 139* (HRCB); idem, 4.XII.2019, bud, fl., *L.S. Santos et al. 207* (HRCB).

Chrysobalanaceae

Couepia grandiflora (Mart. & Zucc.) Benth.: BRASIL. SÃO PAULO: Corumbataí, 18.XI.1961, *H. Amaral* (HRCB 1489); idem, 12.IX.1985, bud, *S.N. Pagano 680* (HRCB); idem, 19.X.1988, fl., *L.C. Saraiva* 54 (HRCB).

Leptobalanus humilis (Cham. & Schltdl.) Sothers & Prance: BRASIL. SÃO PAULO: Corumbataí, 21.VIII.1984, fl., *S.N. Pagano 616* (HRCB).

Connaraceae

Connarus suberosus Planch.: BRASIL. SÃO PAULO: Corumbataí, 25.V.1962, fr., *H. Amaral* (HRCB 1257); Reserva de Cerrado, 20.IV.1963, fr., *C. Moura* (HRCB 1258); 6.X.1983, bud, fl., *M.J.O. Campos 112* (HRCB); idem, 21.VIII.1985, fl., *A. Feddersen Jr 4* (HRCB); idem, Reserva de Cerrado da UNESP, 30.IX.2019, bud, fl., *L.S. Santos & & T.S. El Hindi 196* (HRCB).

Dilleniaceae

Davilla elliptica A.St.-Hil.: BRASIL. SÃO PAULO: Corumbataí, Reserva de Cerrado, 19.VII.1962, *H. Amaral* (HRCB 1266); idem, Reserva de Cerrado da UNESP, 13.VI.2019, fl., *L.S. Santos et al. 68* (HRCB).

Davilla rugosa Poir.: BRASIL. SÃO PAULO: Corumbataí, cerrado de Corumbataí, 3.X.1983, fr., *O. Cesar* (HRCB 3658); Corumbataí, 21.VIII.1984, fr., *A. Feddersen Jr 7* (HRCB); idem, Corumbataí, 31.VIII.1984, fl., *S.N. Pagano 635* (HRCB); idem, Reserva de Cerrado da UNESP, 18.V.2019, bud, fl., *L.S. Santos et al. 35* (HRCB); idem, 18.V.2019, fl., *L.S. Santos et al. 53* (HRCB).

Ebenaceae

Diospyros lasiocalyx (Mart.) B.Walln: BRASIL. SÃO PAULO: Corumbataí, 31.VIII.1984, fl., *S.N. Pagano 623* (HRCB).

Erythroxylaceae

Erythroxylum campestre A.St.-Hil.: BRASIL. SÃO PAULO: Corumbataí, 18.VIII.1983, fl., *M.J.O. Campos 110* (HRCB); idem, 23.IX.1987, fl. & fr., *A. Furlan 356* (HRCB).

Erythroxylum cuneifolium (Mart.) O.E.Schulz: BRASIL. SÃO PAULO: Corumbataí, Reserva de cerrado da FAPESP, 13.IV.1982, fl., *O. Cesar* (HRCB 2436); idem, Cerrado de Corumbataí, 26.X.1982, fl., *M.J.O. Campos 38* (HRCB); idem, Área de Proteção Ambiental (UNESP); idem, 9.XI.2001, fr., *A.C. Bieras 14* (HRCB); idem, Reserva de Cerrado, n.d., fl., *n.d.* (HRCB 1278); Reserva de Cerrado da UNESP, 18.V.2019, fr., *L.S. Santos et al. 1* (HRCB); idem, 4.XII.2019, fr., *P.L.R. Moraes et al 5522* (HRCB); idem, 12.II.2020, bud, fl., *L.S. Santos & M.M. Suemitsu 136* (HRCB); idem, 29.X.2020, bud, fl., *L.S. Santos et al. 275* (HRCB).

Erythroxylum daphnites Mart.: BRASIL. SÃO PAULO: Corumbataí, Reserva de Cerrado, 15.I.1961, bud, fl., *H. Amaral* (HRCB 1272); Reserva de Cerrado, 3.VII.1962, bud, fl., *H. Amaral* (HRCB 1271).

Erythroxylum deciduum A.St.-Hil.: BRASIL. SÃO PAULO: Corumbataí, 14.VII.1965, bud, fl., *H. Vitti* (HRCB 1277).

Erythroxylum pelleterianum A.St.-Hil.: BRASIL. SÃO PAULO: Corumbataí, Reserva de Cerrado, 25.VI.1962, fl. & fr., *H. Vitti* (HRCB 1270); idem, 24.VIII.1982, fl., *O. Cesar* & *S.N. Pagano* 48 (HRCB); idem, 31.VIII.1984, fl., *S.N. Pagano 620* (HRCB); idem, Reserva de Cerrado da UNESP, 11.XII.2019, fr., *L.S. Santos & L.P. Elias 133* (HRCB).

Erythroxylum suberosum A.St.-Hil.: BRASIL. SÃO PAULO: Corumbataí, Reserva de Cerrado, 15.I.1961, fr., *H. Amaral* (HRCB 1275); Reserva de Cerrado, 24 .IX.1961, fl. & fr., *n.d.* (HRCB 1274); idem, 14.V.1965, fl., *H. Vitti* (HRCB 1276); idem, 14.VIII.1967, fr., *H. Vitti* (HRCB 1273); Área de Proteção Ambiental (UNESP); idem, 9.XI.2001, fr., *A.C. Bieras 15* (HRCB).

Euphorbiaceae

Alchornea glandulosa Poepp. & Endl.: BRASIL. SÃO PAULO: Corumbataí, Reserva de Cerrado da UNESP, 18.V.2021, bud, fl., *L.S. Santos et al. 345* (HRCB).

Croton gnaphaloides Schrad.: BRASIL. SÃO PAULO: Corumbataí, Reserva de Cerrado, 6.II.1976, fl., *G. Marinis & O. Cesar 6* (HRCB); idem, 29.III.1976, fl., *G. Marinis & O. Cesar 15* (HRCB); idem, 25.I.1984, fl., *J.A. Mendes* (HRCB 3989); idem, 24.II.2000, fl., *C.E. Carneiro et al. 20* (HRCB); idem, Reserva de Cerrado da UNESP, 12.III.2020, fl., *L.S. Santos & G.G. Queiros 167* (HRCB); idem, 11.III.2021, fl. & fr., *L.S. Santos et al. 320* (HRCB).

Microstachys serrulata (Mart.) Müll.Arg.: BRASIL. SÃO PAULO: Corumbataí, 20.II.1978, fr., *J.C. Toledo 16* (HRCB); idem, 21.VIII.1982, fr., *M.J.O. Campos 48* (HRCB); idem, 7.III.1985, fr., *L. Cordeiro 18* (HRCB).

Sapium glandulosum (L.) Morong: BRASIL. SÃO PAULO: Corumbataí, Mata da UNESP, 2.II.1994, fr., *K.D. Barreto et al.* 1943 (HRCB); idem, Reserva de Cerrado, 23.IV.1962, bud, fl., *H. Amaral* (HRCB 1291); Reserva de Cerrado, 25.VIII.1964, bud, fl. & fr., *H. Vitti* (HRCB 1290); n.d., fl. & fr., *n.d.* (HRCB 1287); Reserva de Cerrado, 16.VIII.1960, bud, fl., *H. Amaral* (HRCB 1289); idem, Reserva de Cerrado da UNESP, 11.XII.2019, bud, fl., *L.S. Santos & L.P. Elias 230* (HRCB).

Fabaceae

Anadenanthera peregrina (L.) Speg.: BRASIL. SÃO PAULO: Corumbataí, Reserva de Cerrado, 24.VIII.1977, bud, fl. & fr., *J.C. Toledo* (HRCB 1342); idem, Cerrado de Corumbataí, 8.IX.1983, fl., *L. Cordeiro* (HRCB 3355); idem, 3.IV.1985, fr., *A. Furlan & O. Cesar 240* (HRCB); idem, 2.IX.1989, bud, fl., *L.C. Saraiva 70* (HRCB); idem, Reserva de Cerrado da UNESP, 18.V.2019, fr., *L.S. Santos et al. 24* (HRCB); idem, 13.VI.2019, fr., *L.S. Santos et al. 81* (HRCB); idem, 11.IX.2019, bud, fl., *L.S. Santos & G.G. Queiros 103* (HRCB); idem, 30.IX.2019, bud, fl., *L.S. Santos & T.S. El Hindi 185* (HRCB); idem, 8.IV.2021, fr., *L.S. Santos et al. 327* (HRCB).

Andira humilis Mart. ex Benth.: BRASIL. SÃO PAULO: Corumbataí, Reserva de Cerrado, 24.VIII.1961, bud, fl., *H. Amaral* (HRCB 1344); idem, 10.X.1985, bud, fl., *A.R. Bueno et al.* (HRCB 7172).

Andira vermifuga Mart. ex Benth.: BRASIL. SÃO PAULO: Corumbataí, Reserva de Cerrado, 30.VIII.1961, bud, fl., *H. Amaral* (HRCB 1355); Reserva de Cerrado, 5.VIII.1964, bud, fl., *H. Vitti* (HRCB 1349); Reserva de

Cerrado, 2.X.1986, bud, fl., *O.A. Silva* (HRCB 7152); Reserva de Cerrado da UNESP, 20.XI.2020, fr., *L.S. Santos & L.P. Elias 291* (HRCB).

Andira cf. *vermifuga* Mart. ex Benth.: BRASIL. SÃO PAULO: Corumbataí, Reserva de Cerrado, 16.III.1963, fr., *H. Vitti* (HRCB 1352).

Bauhinia brevipes Vogel: BRASIL. SÃO PAULO: Corumbataí, Reserva de Cerrado, 3.X.1983, bud, fl. & fr., *O. Cesar* (HRCB 3510); Corumbataí, 7.V.1985, bud, *L. Cordeiro 30* (HRCB); idem, Corumbataí, 14.VI.1983, bud, fl. & fr., *M.J.O. Campos 94* (HRCB); idem, Reserva de Cerrado da UNESP, 13.VI.2019, bud, fl., *L.S. Santos et al. 76* (HRCB); idem, 1.VIII.2019, fl. & fr., *L.S. Santos et al. 147* (HRCB); idem, 16.VI.2021, bud, fl. & fr., *L.S. Santos et al. 366* (HRCB); idem, 6.VII.2021, bud, fl. & fr., *L.S. Santos et al. 373* (HRCB).

Bauhinia holophylla (Bong.) Steud.: BRASIL. SÃO PAULO: Corumbataí, Reserva de Cerrado, 22.XI.1960, bud, fr., *K. Arens* (HRCB 1321); Reserva de Cerrado, 22.II.1965, fl. & fr., *H. Vitti* (HRCB 1323); Corumbataí, 7.V.1985, fr., *L. Cordeiro 29* (HRCB); idem, Reserva de Cerrado da UNESP, 13.VII.2019, fr., *L.S. Santos et al. 109* (HRCB); idem, 29.I.2020, fl., *L.S. Santos & M.N. Saka 270* (HRCB).

Cerradicola cf. *decumbens* (Benth.) L.P.Queiroz: BRASIL. SÃO PAULO: Corumbataí, 9.II.1984, bud, fl., *O. Cesar 154* (HRCB); idem, 27.III.1985, fr., *L. Cordeiro 21* (HRCB); idem, 14.V.1985, fr., *L. Cordeiro 27* (HRCB).

Chamaecrista cathartica (Mart.) H.S.Irwin & Barneby: BRASIL. SÃO PAULO: Corumbataí, Reserva de Cerrado, 20.II.1978, fl. & fr., *J.C. Toledo* (HRCB 1333); idem, 30.VIII.1982, fl. & fr., *M.J. Campos 21* (HRCB); idem, 9.II.1984, fl., *O. Cesar 150* (HRCB); idem, 15.VIII.1984, fl. & fr., *Turma Biologia* (HRCB 4658).

Chamaecrista desvauxii (Collad.) Killip: BRASIL. SÃO PAULO: Corumbataí, Cerrado de Corumbataí, 22.II.1983, bud, fl. & fr., *C.M. Beltrati 2* (HRCB); idem, 9.II.1984, fl. & fr., *O. Cesar 151* (HRCB); idem, 10.IV.1984, fr., *L. Cordeiro 1* (HRCB).

Chamaecrista flexuosa (L.) Greene: BRASIL. SÃO PAULO: Corumbataí, 8.III.1983, fl. & fr., *M.J.O. Campos 81* (HRCB); idem, Cerrado de Corumbataí, 10.XI.1983, bud, fl. & fr., *L. Cordeiro* (HRCB 3791); Corumbataí, 1.XII.1983, fl., *L. Cordeiro* (HRCB 3814); idem, 29.II.1984, bud, fl. & fr., *J.A. Mendes 4* (HRCB); idem, 10.IV.1984, fl., *L. Cordeiro 3* (HRCB); idem, Corumbataí, 29.III.2000, fl., *L. Cordeiro 1* (HRCB); idem, Reserva de Cerrado da UNESP, 12.I.2020, fl. & fr., *L.S. Santos & M.M. Suemitsu 98* (HRCB); idem, 26.I.2021, bud, fl., *L.S. Santos & L.P. Elias 312* (HRCB); idem, 18.V.2021, bud, fl., *L.S. Santos et al. 357* (HRCB).

Chamaecrista rotundifolia (Pers.) Greene: BRASIL. SÃO PAULO: Corumbataí, Reserva de Cerrado, 17.III.1961, fl. & fr., *H. Amaral* (HRCB 1330); idem, 29.III.2000, fl. & fr., *L. Cordeiro et al. 2* (HRCB); idem, Reserva de Cerrado, n.d., fl. & fr., *n.d.* (HRCB 1329).

Clitoria densiflora (Benth) Benth.: BRASIL. SÃO PAULO: Corumbataí, 16.VI.1961, fl., *H. Amaral* (HRCB 1354); n.d., fl., *n.d.* (HRCB 1335).

Copaifera langsdorffii Desf.: BRASIL. SÃO PAULO, Corumbataí, Reserva de Cerrado da UNESP, 4.XII.2019, bud, *P.L.R. Moraes et al. 5538* (HRCB); idem, 11.XII.2019, bud, fl., *L.S. Santos & L.P. Elias 234* (HRCB); idem, 12.XII.2020, fr., *L.S. Santos & M.M. Suemitsu 69* (HRCB); idem, 29.X.2020, fr., *L.S. Santos et al. 276* (HRCB); idem, 26.I.2021, bud, fl., *L.S. Santos & L.P. Elias 303* (HRCB); idem, 7.VII.2021, fr., *L.S. Santos et al. 374* (HRCB).

Crotalaria maypurensis Kunth: BRASIL. SÃO PAULO: Corumbataí, Cerrado de Corumbataí, 29.III.1983, fl., *M.J.O. Campos 70* (HRCB); idem, 25.I.1984, fl., *J.A. Mendes* (HRCB 3994).

Crotalaria micans Link: BRASIL. SÃO PAULO, Corumbataí, Reserva de Cerrado da UNESP, 29.I.2020, bud, fl., *L.S. Santos & M.N. Saka 264* (HRCB); idem, 26.I.2021, bud, fr., *L.S. Santos & L.P. Elias 315* (HRCB).

Crotalaria pallida Aiton var. *obovata* (G.Don) Polhill: BRASIL. SÃO PAULO: Corumbataí, 29.III.2000, bud, fl. & fr., *L. Cordeiro et al. 5* (HRCB).

Dahlstedtia muehlbergiana (Hassl.) M.J.Silva & A.M.G.Azevedo: BRASIL. SÃO PAULO: Corumbataí, 1.XII.1999, bud, fl., *M.A. Assis & V.T. Rampin 1332* (HRCB).

Dalbergia frutescens (Vell.) Britton var. *frutescens*: BRASIL. SÃO PAULO: Corumbataí, Reserva de Cerrado, 6.V.1961, bud, fl., *H. Amaral* (HRCB 1348).

Dalbergia miscolobium Benth.: BRASIL. SÃO PAULO: Corumbataí, 25.I.1984, bud, fl., *J.A. Mendes* (HRCB 3998); idem, Reserva de Cerrado da UNESP, 13.VI.2019, fr., *L.S. Santos et al. 75* (HRCB); idem, 12.II.2020, fr., *L.S. Santos & M.M. Suemitsu 118* (HRCB); idem, 26.I.2021, bud, fl., *L.S. Santos & L.P. Elias 302* (HRCB).

Desmodium tortuosum (Sw.) DC.: BRASIL. SÃO PAULO: Corumbataí, 29.III.2000, fr., *L. Cordeiro et al. 3* (HRCB).

Dimorphandra mollis Benth.: BRASIL. SÃO PAULO: Corumbataí, Reserva de Cerrado, 29.XI.1961, fl., *H. Amaral* (HRCB 1346); idem, 21.V.1984, fr., *S.N. Pagano 626* (HRCB); idem, 13.VI.2019, fr., *L.S. Santos et al. 77* (HRCB); idem, 4.XII.2019, bud, fl., *P.L.R. Moraes et al.* 5526 (HRCB); idem, 11.XII.2019, fl., *L.S. Santos & L.P. Elias 223* (HRCB); idem, 16.VI.2021, fr., *L.S. Santos et al. 365* (HRCB).

Indigofera hirsuta L.: BRASIL. SÃO PAULO: Corumbataí, 29.III.2000, fl., *L. Cordeiro et al. 4* (HRCB).

Leptolobium dasycarpum Vogel: BRASIL. SÃO PAULO: Corumbataí, 3.VII.1983, bud, fl., *M.J.O. Campos 73* (HRCB); idem, 4.IV.1984, bud, fl., *L. Cordeiro 8* (HRCB); idem, Corumbataí, 3.IV.1985, fr., *A. Furlan & O. Cesar 238* (HRCB); idem, 2.XII.1997, bud, fl., *M.A. Assis et al.* 1078 (HRCB); idem, Reserva de Cerrado da UNESP, 4.XII.2019, fl., *L.S. Santos et al. 217* (HRCB); idem, 29.I.2020, fr., *L.S. Santos & M.N. Saka 261* (HRCB); idem, 29.X.2020, fr., *L.S. Santos et al. 280* (HRCB); idem, 26.I.2021, fl., *L.S. Santos & L.P. Elias 309* (HRCB).

Leptolobium elegans Vogel: BRASIL. SÃO PAULO: Corumbataí, Reserva de Cerrado da UNESP, 4.XII.2019, fl., *L.S. Santos et al. 213* (HRCB); idem, 4.XII.2019, bud, fl., *P.L.R. Moraes et al. 5528* (HRCB); idem, 11.XII.2019, fl. & fr., *L.S. Santos & L.P. Elias 228* (HRCB); idem, 29.I.2020, fr., *L.S. Santos & M.N. Saka 262* (HRCB); idem, 12.II.2020, fr., *L.S. Santos & M.M. Suemitsu 224* (HRCB); idem, 29.X.2020, bud, fl., *L.S. Santos et al. 279* (HRCB); idem, 20.XI.2020, bud, fl. & fr., *L.S. Santos & L.P. Elias 292* (HRCB); idem, 26.I.2021, fr., *L.S. Santos & L.P. Elias 296* (HRCB).

Machaerium acutifolium Vogel: BRASIL. SÃO PAULO: Corumbataí, Reserva de Cerrado, 10.XI.1988, bud, fl., *L.C. Saraiva 56* (HRCB); idem, 26.X.1994, bud, fl., *Adriana 7* (HRCB); idem, 24.II.2000, fr., *V.B. Ziparro et al. 1895* (HRCB); idem, Reserva de Cerrado da UNESP, 4.XII.2019, fl., *L.S. Santos et al. 218* (HRCB); idem, 4.XII.2019, fr., *L.S. Santos et al. 219* (HRCB); idem, 4.XII.2019, fr., *P.L.R. Moraes et al. 5527* (HRCB); idem, 29.X.2020, bud, *L.S. Santos et al. 286* (HRCB).

Machaerium nyctitans (Vell.) Benth.: BRASIL. SÃO PAULO: Corumbataí, Reserva de Cerrado da UNESP, 1.VIII.2019, fr., *L.S. Santos et al. 134* (HRCB); idem, 11.III.2021, bud, fl., *L.S. Santos et al. 318* (HRCB); idem, 16.VI.2021 fr., *L.S. Santos et al. 370* (HRCB).

Machaerium villosum Vogel: BRASIL. SÃO PAULO: Corumbataí, Reserva de Cerrado, 14.VIII.1963, *H. Vitti* (HRCB 1350).

Mimosa debilis Humb. & Bonpl. ex Willd.: BRASIL. SÃO PAULO: Corumbataí, Reserva de Cerrado, 20.II.1978, bud, fl., *J.C. Toledo* (HRCB 1341); idem, 10.IV.1984, bud, fl. & fr., *L. Cordeiro 2* (HRCB); idem, Reserva de Cerrado da UNESP, 12.II.2020, bud, fl., *L.S. Santos & M.M. Suemitsu 82* (HRCB).

Mimosa xanthocentra Mart. var. *subsericea* (Benth.) Barneby: BRASIL. SÃO PAULO: Corumbataí, 25.I.1984, bud, fl., *J.A. Mendes* (HRCB 3981); idem, 24.II.2000, fl., *C.E. Carneiro et al. 34* (HRCB); idem, 29.III.2000, fl. & fr., *L. Cordeiro et al. 1* (HRCB); idem, Reserva de Cerrado da UNESP, 8.IV.2021, fr., *L.S. Santos et al. 334* (HRCB).

Senegalia lowei (L.Rico) Seigler & Ebinger: BRASIL. SÃO PAULO: Corumbataí, 16.XI.1959, bud, fl., *K. Arens* (HRCB 1337); n.d., bud, fl., *n.d.* (HRCB 1340).

Senna pendula (Humb. & Bonpl. ex Willd.) H.S.Irwin & Barneby: BRASIL. SÃO PAULO: Corumbataí, 25.III.2002, bud, fl., *V.T. Rampin 1538* (HRCB); idem, 18.V.2019, fr., *L.S. Santos et al. 7* (HRCB); idem, 8.IV.2021, bud, fl., *L.S. Santos et al. 336* (HRCB).

Senna rugosa (G.Don) H.S.Irwin & Barneby: BRASIL. SÃO PAULO: Corumbataí, Reserva de Cerrado, 7.XII.1960, bud, fl., *Benedito & H. Amaral* (HRCB 1324); Reserva de Cerrado, 20.II.1978, bud, fl., *J.C. Toledo* (HRCB 1327); 9.II.1984, bud, fl., *O. Cesar 152* (HRCB); idem, 10.IV.1984, bud, fl., *L. Cordeiro 6* (HRCB); idem, 12.II.2020, bud, fl., *L.S. Santos & M.M. Suemitsu 235* (HRCB); idem, 11.III.2021, fl., *L.S. Santos et al. 316* (HRCB); idem, 8.IV.2021, fl., *L.S. Santos et al. 332* (HRCB); idem, 8.IV.2021, fl. & fr., *L.S. Santos et al. 337* (HRCB); idem, 18.V.2021, fl. & fr., *L.S. Santos et al. 352* (HRCB).

Stryphnodendron adstringens (Mart.) Coville: BRASIL. SÃO PAULO: Corumbataí, Reserva de Cerrado, 12.IX.1960, fl., *H. Amaral* (HRCB 1345); idem, Cerrado de Corumbataí, 31.VIII.1983, bud, fl., *I.V. Pereira 1*

(HRCB); idem, Corumbataí, 15.VIII.1984, fl., *Turma Biologia* (HRCB 4660); Corumbataí, 19.X.1988, bud, fl., *L.C. Saraiva 53* (HRCB); idem, Corumbataí, n.d., fl., *n.d.* (HRCB 1343).

Stryphnodendron rotundifolium Mart.: BRASIL. SÃO PAULO: Corumbataí, 17.X.1997, bud, fl., *O. Malaspina & P. Cintra* (HRCB 27392); idem, Cerrado de Corumbataí, 10.I.1982, bud, fl., *M.J.O. Campos 68* (HRCB); idem, Cerrado de Corumbataí, 8.IX.1983, fr., *L. Cordeiro* (HRCB 3354); Reserva de Cerrado, 15.IX.1960, bud, *H. Amaral* (HRCB 1338); Reserva de Cerrado da UNESP, 1.VIII.2019, fr., *L.S. Santos et al. 154* (HRCB); idem, 4.XII.2019, bud, fl., *L.S. Santos et al. 216* (HRCB); idem, 4.XII.2019, bud, fl., *P.L.R. Moraes et al. 5529* (HRCB); idem, 4.XII.2019, *P.L.R. Moraes et al. 5531* (HRCB); idem, 16.VI.2021, fr., *L.S. Santos et al. 369* (HRCB); idem, 6.VII.2021, fr., *L.S. Santos et al. 340* (HRCB).

Stylosanthes scabra Vogel: BRASIL. SÃO PAULO: Corumbataí, 15.VIII.1984, *Turma Biologia* (HRCB 4668).

Stylosanthes viscosa (L.) Sw.: BRASIL. SÃO PAULO: Corumbataí, 10.IV.1984, fl., *L. Cordeiro 5* (HRCB).

Lacistemataceae

Lacistema hasslerianum Chodat: BRASIL. SÃO PAULO: Corumbataí, Cerrado de Corumbataí, 30.VI.1981, fl., *O. Cesar & S.N. Pagano 28* (HRCB); idem, 24.VIII.1982, fl., *O. Cesar & S.N. Pagano 49* (HRCB). Corumbataí, 31.VIII.1984, bud, *S.N. Pagano 605* (HRCB); idem, 31.VIII.1984, bud, fl., *S.N. Pagano 613* (HRCB); idem, 22.X.1984, fr., *J.A. Mendes 25* (HRCB); idem, 23.IX.1987, fl., *A. Furlan 354* (HRCB); idem, 16.X.1996, fr., *A.L. Celia 2* (HRCB); idem, Reserva de Cerrado da UNESP, 11.IX.2019, fl., *L.S. Santos & G.G. Queiros 124* (HRCB); idem, 14.VIII.2019, bud, *L.S. Santos et al. 160* (HRCB); idem, 4.XII.2019, fr., *L.S. Santos et al. 201* (HRCB); idem, 20.XI.2020, fr., *L.S. Santos & L.P. Elias 295* (HRCB).

Lamiaceae

Aegiphila integrifolia (Jacq.) Moldenke: BRASIL. SÃO PAULO, Corumbataí, Reserva de Cerrado da UNESP, 17.XII.2019, bud, fl., *L.S. Santos & L.P. Elias 244* (HRCB); idem, 4.XII.2019, bud, fl., *P.L.R. Moraes et al. 5520* (HRCB).

Aegiphila verticillata Vell.: BRASIL. SÃO PAULO, Corumbataí, Reserva de Cerrado da UNESP, 30.IX.2019, bud, fl., *L.S. Santos & T.S. El Hindi 190* (HRCB).

Cyanocephalus lippioides (Pohl ex Benth.) Harley & J.F.B.Pastore: BRASIL. SÃO PAULO: Corumbataí, Reserva de Cerrado, 7.V.1961, bud, fl., *H. Amaral* (HRCB); idem, 6.VII.1962, bud, fl., *n,d.* (HRCB 1309); 4.V.1984, bud, fl., *L.C. Saraiva 22* (HRCB); idem, 21.VIII.1985, bud, fl., *A. Feddersen Jr. 2* (HRCB); idem, 18.X.1986, fl., *A. Brieger* (HRCB 7089).

Medusantha eriophylla (Pohl ex Benth.) Harley & J.F.B.Pastore: BRASIL. SÃO PAULO: Corumbataí, Cerrado de Corumbataí, 8.III.1983, fl., *M.J.O. Campos 80* (HRCB); idem, Corumbataí, 25.I.1984, fl., *J.A. Mendes* (HRCB 3996); 4.III.1985, fl., *L.C. Saraiva 41* (HRCB); idem, Reserva de Cerrado da UNESP, 12.II.2020, bud, fl., *L.S. Santos & M.M. Suemitsu 91* (HRCB).

Medusantha sp.: BRASIL. SÃO PAULO: Corumbataí, Reserva de Cerrado da UNESP, 6.VII.2021, bud, fl., *L.S. Santos et al. 343* (HRCB); idem, 18.V.2021, bud, fl., *L.S. Santos et al. 360* (HRCB).

Lauraceae

Ocotea corymbosa (Meisn.) Mez: BRASIL. SÃO PAULO: Corumbataí, 31.VIII.1984, fr., *S.N. Pagano 611* (HRCB); idem, Reserva de Cerrado da UNESP, 11.XII.2019, bud, fl., *L.S. Santos & L.P. Elias 231* (HRCB); idem, 17.XII.2019, bud, fl., *L.S. Santos & L.P. Elias 238* (HRCB); idem, 17.XII.2019, fl., *L.S. Santos & L.P. Elias 245* (HRCB); idem, 10.I.2020, bud, fl., *L.S. Santos & R.N. Leite 247* (HRCB); idem, 10.I.2020, bud, fl., *L.S. Santos & R.N. Leite 255* (HRCB); idem, 4.XII.2019, bud, fl., *P.L.R. Moraes et al. 5541* (HRCB).

Ocotea pulchella (Nees & Mart.) Mez: BRASIL. SÃO PAULO: Corumbataí, Reserva de Cerrado, 22.XII.1962, fr., *H. Amaral* (HRCB 1320); idem, Cerrado de Corumbataí, 30.VI.1981, fr., *O. Cesar & S.N. Pagano 45* (HRCB); idem, Cerrado de Corumbataí, 29.X.1983, fr., *L.C. Saraiva 29* (HRCB); idem, 3.IV.1984, bud, fl., *M.J.O. Campos 43* (HRCB); idem, 31.VIII.1984, fr., *S.N. Pagano 612* (HRCB); idem, 28.XI.1989, bud, fl., *L.C. Saraiva 75* (HRCB); idem, 29.XI.1989, bud, fl., *L.C. Saraiva 76* (HRCB); idem, Reserva da UNESP, 10.VIII.2006, fr., *J.A. Lombardi et al. 6412* (HRCB); idem, Reserva de Cerrado da UNESP, 18.V.2019, fr., *L.S. Santos et al. 4* (HRCB); idem, 13.VII.2019, bud, fr., *L.S. Santos et al. 115* (HRCB); idem, 14.VIII.2019, fr., *L.S. Santos et al. 176* (HRCB);

idem, 4.XII.2019, bud, fl., *L.S. Santos et al. 221* (HRCB); idem, 11.XII.2019, bud, fl., *L.S. Santos & L.P. Elias 233* (HRCB); idem, 11.XII.2019, bud, fl., *L.S. Santos & L.P. Elias 237* (HRCB); idem, 26.I.2021, bud, fl., *L.S. Santos & L.P. Elias 299* (HRCB); idem, 4.XII.2019, bud, fr., *P.L.R. Moraes et al. 5542* (HRCB).

Persea willdenovii Kosterm.: BRASIL. SÃO PAULO: Corumbataí, Reserva Prof. Dr. Karl Arens, 28.IX.2012, bud, fl., *P.L.R. Moraes 3494* (HRCB); idem, Reserva Prof. Dr. Karl Arens, 20.X.2012, bud, fl., *P.L.R. Moraes 3518* (HRCB); idem, Reserva Prof. Dr. Karl Arens, 7.XII.2012, fr., *P.L.R. Moraes & R.S. Pacheco 3519* (HRCB).

Loganiaceae

Strychnos bicolor Progel: BRASIL. SÃO PAULO: Corumbataí, cerrado de Corumbataí, 30.VI.1981, *O. Cesar & S.N. Pagano 18* (HRCB); idem, Corumbataí, 29.II.1984, bud, *J.A. Mendes 1* (HRCB); idem, 24.II.2000, *V.B. Ziparro et al. 1884* (HRCB); idem, Reserva de Cerrado de Corumbataí, 13.VII.2019, fr., *L.S. Santos et al. 88* (HRCB).

Lythraceae

Cuphea thymoides Cham. & Schltdl.: BRASIL. SÃO PAULO: Corumbataí, Reserva de Cerrado, 1962., fl., *C. Moura* (HRCB 1360).

Diplusodon virgatus Pohl: BRASIL. SÃO PAULO: Corumbataí, Reserva de Cerrado de Corumbataí, 18.V.1977, fl. & fr., *G. Marinis & O. Cesar 18* (HRCB); idem, Reserva de Cerrado da UNESP, 11.V.1983, fl. & fr., *L.C. Saraiva 19* (HRCB); idem, 29.II.1984, fl., *J.A. Mendes 7* (HRCB); idem, 26.III.1985, fl. & fr., *M.J.O. Campos 87* (HRCB); idem, 30.IV.1985, fl. & fr., *L. Cordeiro 32* (HRCB); idem, 27.IV.1990, fl. & fr., *R. Monteiro et al.* (HRCB 11778); idem, Reserva de Cerrado da UNESP, 13.VI.2019, fr., *L.S. Santos et al. 71* (HRCB); idem, 11.XII.2019, fr., *L.S. Santos & L.P. Elias 236* (HRCB); idem, 8.IV.2021, bud, fl., *L.S. Santos et al. 324* (HRCB); idem, 18.V.2021, fl. & fr., *L.S. Santos et al. 359* (HRCB).

Lafoensia pacari A.St.-Hil.: BRASIL. SÃO PAULO: Corumbataí, 24.II.2000, fr., *V.B. Zipparro et al. 1894* (HRCB); idem, Reserva de Cerrado da UNESP, 18.V.2019, *L.S. Santos et al. 6* (HRCB); idem, 1.VIII.2019, fr., *L.S. Santos et al. 123* (HRCB); idem, 4.XII.2019, bud, fl., *L.S. Santos et al. 202* (HRCB).

Malpighiaceae

Banisteriopsis campestris (A.Juss.) Little: BRASIL. SÃO PAULO: Corumbataí, Reserva de Cerrado, 18.X.1962, bud, fl., *H. Amaral* (HRCB 1371).

Banisteriopsis stellaris (Griseb.) B.Gates: BRASIL. SÃO PAULO: Corumbataí, Reserva de Cerrado da UNESP, 18.V.2019, fl. & fr., *L.S. Santos et al. 28* (HRCB); idem, 12.II.2020, bud, fl. & fr., *L.S. Santos & M.M. Suemitsu 55* (HRCB); idem, 1.VIII.2019, bud, fl., *L.S. Santos et al. 149* (HRCB); idem, 11.III.2021, bud, fl., *L.S. Santos et al. 317* (HRCB).

Byrsonima coccolobifolia Kunth: BRASIL. SÃO PAULO: Corumbataí, Reserva de Cerrado, 16.XI.1959, bud, *K. Arens* (HRCB 1383); Reserva de Cerrado, 12.X.1962, bud, fl., *H. Amaral* (HRCB 1378); idem, 20.X.1983, bud, fl., *M.J.O. Campos 34* (HRCB); idem, Cerrado de Corumbataí, 3.XI.1983, bud, fl., *L. Cordeiro* (HRCB 3530); 4.XII.2019, bud, *Moraes, PL.R. 5536* (HRCB).

Byrsonima intermedia A.Juss.: BRASIL. SÃO PAULO: Corumbataí, Reserva de Cerrado, 12.IX.1962, bud, *H. Amaral* (HRCB 1373); Reserva de Cerrado, 12.X.1962, bud, fl., *H. Amaral* (HRCB 1379); Reserva de Cerrado, 20.II.1978, bud, *J.C. Toledo* (HRCB 1369); idem, 28.XII.1983, bud, fl., *J.A. Mendes* (HRCB 3984); idem, 11.VII.1984, fl. & fr., *C.M. Beltrati 68* (HRCB); idem, 3.IV.1985, fr., *A. Furlan & O. Cesar 241* (HRCB); idem, 28.XI.1989; fl., *L.C. Saraiva 74* (HRCB); idem, 28.X.999; fl. & fr., *T.L. Bisi* (HRCB 33053); Reserva de Cerrado da UNESP, 18.V.2019, fr., *L.S. Santos et al. 18* (HRCB); idem, 1.VIII.2019, fr., *L.S. Santos et al. 144* (HRCB); idem, 30.IX.2019, fr., *L.S. Santos & T.S. El Hindi 183* (HRCB); idem, 4.XII.2019, bud, fl. & fr., *L.S. Santos et al. 208* (HRCB); idem, 4.XII.2019, bud, fl. & fr., *L.S. Santos et al. 209* (HRCB); idem, 29.X.2020, bud, fl., *L.S. Santos et al. 278* (HRCB); idem, 26.I.2021, fr., *L.S. Santos & L.P. Elias 301* (HRCB); idem, 26.I.2021, bud, fl., *L.S. Santos & L.P. Elias 304* (HRCB).

Byrsonima verbascifolia (L.) DC.: BRASIL. SÃO PAULO: Corumbataí, Reserva de Cerrado, 16.XI.1959, fr., *K. Arens* (HRCB 1381); idem, 2.X.1963, bud, fl., *H. Amaral* (HRCB 1367); Corumbataí, 24.II.2000, fr., *C.E.*

Carneiro et al. 26 (HRCB); idem, Área de proteção ambiental sob responsabilidade da UNESP, 24.II.2000, fr., *V.F.O. Miranda et al. 164* (HRCB).

Heteropterys umbellata A.Juss.: BRASIL. SÃO PAULO: Corumbataí, Reserva de Cerrado, 14.IX.1962, fl. & fr., *H. Amaral* (HRCB 1375); 4.V.1983, fl. & fr., *L.C. Saraiva 37* (HRCB); idem, 9.II.1984, fl. & fr., *O. Cesar 147* (HRCB).

Peixotoa tomentosa A.Juss.: BRASIL. SÃO PAULO: Corumbataí, Reserva de Cerrado, 7.X.1962, fl., *H. Amaral* (HRCB 1376); Reserva de Cerrado da UNESP, 13.VII.2019, fl., *L.S. Santos et al. 119* (HRCB).

Malvaceae

Byttneria sagittifolia A.St.-Hil.: BRASIL. SÃO PAULO: Corumbataí, Reserva de Cerrado, 6.VIII.1964, fl., *H. Amaral* (HRCB 1593).

Eriotheca gracilipes (K.Schum.) A.Robyns: BRASIL. SÃO PAULO: Corumbataí, 31.VIII.1984, bud, fl., *S.N. Pagano 609* (HRCB); idem, 6.VII.1989, bud, fl., *L.C. Saraiva 69* (HRCB); idem, Reserva de Cerrado da UNESP, 13.VII.2019, bud, fl., *L.S. Santos et al. 114* (HRCB); idem, 14.VIII.2019, bud, fr., *L.S. Santos et al. 179* (HRCB); idem, 29.X.2020, fr., *L.S. Santos et al. 285* (HRCB).

Luehea grandiflora Mart.: BRASIL. SÃO PAULO: Corumbataí, Reserva de Cerrado da UNESP, Borda em fragmento de cerradão, 18.XI.2023, fr., *L.S. Santos 378* (HRCB).

Pavonia malacophylla (Link & Otto) Garcke: BRASIL. SÃO PAULO: Corumbataí, 7.V.1984, bud, fl. & fr., *L. Cordeiro 10* (HRCB).

Melastomataceae

Leandra aurea (Cham.) Cogn.: BRASIL. SÃO PAULO: Corumbataí, 28.XII.1983, fr., *J.A. Mendes* (HRCB 3999); idem, 2.IX.1989, fl., *L.C. Saraiva 72* (HRCB).

Miconia albicans (Sw.) Triana: BRASIL. SÃO PAULO: Corumbataí, Cerrado de Corumbataí, 30.VI.1981, bud, *O. Cesar & S.N. Pagano 31* (HRCB); idem, Corumbataí, 10.IX.1982, bud, fl., *M.J.O. Campos* (HRCB 6480); idem, 21.V.1984, bud, fl., *J.A. Mendes 12* (HRCB); idem, 15.VIII.1984, bud, fl., *Turma-Biologia* (HRCB 4657); idem, 2.IX.1989, fl., *L.C. Saraiva 71* (HRCB); idem, Reserva de Cerrado da UNESP, 17.XII.2019, fr., *L.S. Santos & L.P. Elias 243* (HRCB).

Miconia fallax DC: BRASIL. SÃO PAULO: Corumbataí, 10.IX.1982, fl., *M.J. Campos 27* (HRCB); idem, 26.X.1982, bud, fl., *M.J.O. Campos 36* (HRCB).

Miconia flammea Casar. BRASIL. SÃO PAULO: Corumbataí, 17.XI.1983, bud, *Laurié* (HRCB 3822); idem, 10.XII.1983, bud, fl., *M.J.O. Campos 123* (HRCB); idem, 21.II.1984, fr., *J.A. Mendes 10* (HRCB); idem, Reserva de Cerrado da UNESP, 18.V.2019, fr., *L.S. Santos et al. 44* (HRCB); idem, 4.XII.2019, bud, fl., *L.S. Santos et al. 206* (HRCB); idem, 10.I.2020, fr., *L.S. Santos & R.N. Leite 254* (HRCB).

Miconia ligustroides (DC.) Naudin: BRASIL. SÃO PAULO: Corumbataí, Cerrado de Corumbataí, 20.II.1978, fr., fl., *J.C. Toledo* (HRCB 1389); idem, 30.VI.1981, fr., *O. Cesar & S.N. Pagano 27* (HRCB); idem, 21.XII.1982, fl. & fr., *M.J.O. Campos 52* (HRCB); idem, Corumbataí, 25.I.1984, fl. & fr., *J.A. Mendes* (HRCB 3988); idem, 17.V.1984, *S.N. Pagano 633b* (HRCB); idem, Corumbataí, 29.XI.1989, bud, fl., *L.C. Saraiva 77* (HRCB); idem, 16.II.1990, fl. & fr., *L.C. Saraiva 81* (HRCB); idem, Reserva de Cerrado,18.I.1991, bud, fl. & fr., *L.C. Saraiva 86* (HRCB); idem, Corumbataí, 24.II.2000, fl., *C.E. Carneiro et al. 35* (HRCB); idem, Reserva de Cerrado da UNESP, 18.V.2019, bud, fr., *L.S. Santos et al. 57* (HRCB); idem, 13.VII.2019, fr., *L.S. Santos et al. 110* (HRCB); idem, 1.VIII.2019, fl. & fr., *L.S. Santos et al. 146* (HRCB); idem, 14.VIII.2019, bud, fl. & fr., *L.S. Santos et al. 169* (HRCB); idem, 17.XII.2019, fl., *L.S. Santos & L.P. Elias 246* (HRCB); idem, 26.I.2021, bud, fl. & fr., *L.S. Santos & L.P. Elias 311* (HRCB); idem, 18.V.2021, bud, fl. & fr., *L.S. Santos et al. 354* (HRCB); idem, 16.VI.2021, fr., *L.S. Santos et al. 362* (HRCB); idem, 4.XII.2019, bud, fl. & fr., *P.L.R. Moraes et al. 5534* (HRCB).

Miconia paucidens DC.: BRASIL. SÃO PAULO: Corumbataí, 17.V.1984, bud, *S.N. Pagano 633* (HRCB).

Miconia rubiginosa (Bonpl.) DC.: BRASIL. SÃO PAULO: Corumbataí, Cerrado de Corumbataí, 25.VIII.1980, fl. & fr., *O. Cesar & S.N. Pagano 46* (HRCB); idem, 28.XII.1983, bud, fl., *J.A. Mendes* (HRCB 3980); 7.III.1985, bud, fl., *M.J.O. Campos 135* (HRCB); idem, 29.XI.1989, bud, fl., *L.C. Saraiva 78* (HRCB); idem, 29.XI.1989, fl., *L.C. Saraiva 79* (HRCB); idem, 24.II.2000, fl., *C.E. Carneiro et al. 25* (HRCB); idem, 24.II.2000, fr., *C.E.*

Carneiro et al. 21 (HRCB); idem, 24.II.2000, Área de Proteção Ambiental (UNESP); idem, fl., *C. Reis 8* (HRCB); idem, Área de proteção ambiental sob responsabilidade da UNESP, 24.II.2000, fr., *V.F.O. Miranda et al. 172* (HRCB); idem, Reserva de Cerrado da UNESP, 18.V.2019, bud, fl., *L.S. Santos et al. 21* (HRCB); idem, 13.VI.2019, fl. & fr., *L.S. Santos et al. 64* (HRCB); idem, 13.VII.2019, bud, fl. & fr., *L.S. Santos et al. 85* (HRCB); idem, 13.VII.2019, bud, fl. & fr., *L.S. Santos et al. 86* (HRCB); idem, 13.VII.2019, fr., *L.S. Santos et al. 93* (HRCB); idem, 13.VII.2019, bud, fl. & fr., *L.S. Santos et al. 99* (HRCB); idem, 13.VII.2019, fr., *L.S. Santos et al. 102* (HRCB); idem, 1.VIII.2019, fl. & fr., *L.S. Santos et al. 156* (HRCB); idem, 14.VII.2019, fr., *L.S. Santos et al. 163* (HRCB); idem, 17.XII.2019, bud, fl. & fr., *L.S. Santos & L.P. Elias 242* (HRCB); idem, 18.V.2021, bud, fl. & fr., *L.S. Santos et al. 348* (HRCB); idem, 6.VII.2021, bud, fl. & fr., *L.S. Santos et al. 349* (HRCB); idem, 4.XII.2019, bud, fl. & fr., *P.L.R. Moraes et al. 5535* (HRCB); idem, 4.XII.2019, bud, fl., *P.L.R. Moraes et al. 5537* (HRCB).

Miconia sellowiana Naudin: BRASIL. SÃO PAULO: Corumbataí, Cerrado de Corumbataí, 24.VIII.1982, fl., *O. Cesar & S.N. Pagano 52* (HRCB); idem, Corumbataí, 21.VIII.1985, bud, fl., *A. Feddersen Jr. 1* (HRCB); idem, Reserva de Cerrado de UNESP, 11.IX.2019, bot, fl., *L.S. Santos & G.G. Queiros 26* (HRCB); idem, 30.VIII.2019, bud, fl., *L.S. Santos & A.G. Mitri 162* (HRCB); idem, 29.X.2020, fr., *L.S. Santos et al. 288* (HRCB); idem, 4.XII.2019, fr., *P.L.R. Moraes et al. 5519* (HRCB).

Miconia stenostachya DC.: BRASIL. SÃO PAULO: Corumbataí, 24.VIII.1982, bud, fl., *O. Cesar & S.N. Pagano 50* (HRCB); idem, 21.V.1984, bud, fl., *J.A. Mendes 13* (HRCB); idem, 31.VIII.1984, bud, fl., *S.N. Pagano 628* (HRCB); idem, 19.IX.1999, *D.S. Alves* (HRCB 33049); idem, Reserva de Cerrado da UNESP, 18.V.2019, bud, fr., *L.S. Santos et al. 50* (HRCB); idem, 14.VIII.2019, bud, fl., *L.S. Santos et al. 159* (HRCB).

Microlicia polystemma Naudin: BRASIL. SÃO PAULO: Corumbataí, 17.VIII.1961, fl., *H. Amaral* (HRCB 1362).

Pleroma stenocarpum (Schrank & Mart. ex DC.) Triana: BRASIL. SÃO PAULO: Corumbataí, Reserva de Cerrado, 29.III.1976, *G. Marinis* (HRCB 1387); idem, 20.II.1978, *J.C. Toledo* (HRCB 1390) Corumbataí, 8.III.1985, bud, fl. & fr., *M.J.O. Campos 85* (HRCB); idem, .V.1985, *O. Cesar 588* (HRCB).

Meliaceae

Cedrela fissilis Vell.: BRASIL. SÃO PAULO: Corumbataí, Reserva de Cerrado da UNESP, 13.VII.2019, fr., *L.S. Santos et al. 116* (HRCB); idem, 3.V.2021, fr., *L.S. Santos & L.P. Elias 341* (HRCB); idem, 18.V.2021, fr., *L.S. Santos et al. 346* (HRCB).

Guarea guidonia (L.) Sleumer: BRASIL. SÃO PAULO: Corumbataí, Reserva de Cerrado da UNESP, 5.X.2023, fr. L.S. Santos 377 (HRCB).

Moraceae

Ficus guaranitica Chodat: BRASIL. SÃO PAULO: Corumbataí, Reserva de Cerrado da UNESP, 18.V.2019, fl., *L.S. Santos et al. 52* (HRCB); idem, 13.VII.2019, fl., *L.S. Santos et al. 96* (HRCB); idem, 12.II.2020, fl., *L.S. Santos & M.M. Suemitsu 101* (HRCB).

Myristicaceae

Virola sebifera Aubl.: BRASIL. SÃO PAULO: Corumbataí, Cerrado de Corumbataí, 13.VII.1981, fr., *O. Cesar & S.N. Pagano 32* (HRBC); idem, Cerrado de Corumbataí, 31.VIII.1983, fr., *C.S. Barbieri et al. 10* (HRCB); idem, 9.II.1984, bud, fl., *O. Cesar 149* (HRCB); idem, 21.II.1984, bud, fl., *J*.A. Mendes 11 (HRCB); idem, 7.III.1985, fl., *L. Cordeiro 19* (HRCB); idem, 22.III.1989, fl. & fr., *L.C. Saraiva 64* (HRCB); idem, Área de Proteção ambiental sob responsabilidade da UNESP, 24.II.2000, bud, fl., *R. Constantino et al. 174* (HRCB); idem, Reserva da UNESP, 10.VIII.2006, fr., *J.A. Lombardi et al. 6404* (HRCB); idem, Reserva de Cerrado da UNESP, 18.V.2019, fr., *L.S. Santos et al. 43* (HRCB); idem, 13.VI.2019, fr., *L.S. Santos et al. 67* (HRCB); idem, 13.VII.2019, bud, *L.S. Santos et al. 90* (HRCB); idem, 13.VII.2019, fr., *L.S. Santos et al. 112* (HRCB); idem, 16.VI.2021, fr., *L.S. Santos et al. 364* (HRCB).

Myrtaceae

Blepharocalyx salicifolius (Kunth) O.Berg: BRASIL. SÃO PAULO: Corumbataí, 22.X.1984, bud, fl., *J.A. Mendes 23* (HRCB); idem, 19.X.1988, bud, fl., *L.C. Saraiva 51* (HRCB); idem, Reserva de Cerrado da UNESP, 10.I.2020, fr., *L.S. Santos & R.N. Leite 251* (HRCB).

Campomanesia pubescens (Mart. ex DC.) O.Berg: BRASIL. SÃO PAULO: Corumbataí, Reserva de Cerrado, 4.IX.1962, bud, fl., *n.d.* (HRCB 1454); idem, 16.VI.1964, bud, fl., *H. Vitti* (HRCB 1450); idem, 14.IX.1979, fl., *O. Cesar* (HRCB 1098); idem, Cerrado de Corumbataí, 30.IX.1982, bud, fl., *O. Cesar & S.N. Pagano 53* (HRCB); idem, Cerrado de Corumbataí, 31.VIII.1983, fl., *I.V. Pereira 5* (HRCB); idem, 31.VIII.1984, bud, fl., *S.N. Pagano 610* (HRCB); idem, Reserva de Cerrado da UNESP, 30.IX.2019, bud, fl., *L.S. Santos & T.S. El Hindi 184* (HRCB); idem, 29.X.2020, fr., *L.S. Santos et al. 287* (HRCB).

Eugenia aurata O.Berg: BRASIL. SÃO PAULO: Corumbataí, 18.VIII.1983, fl., *MJ.O. Campos 112* (HRCB); idem, 4.IV.1984, bud, fl. & fr., *L. Cordeiro 9* (HRCB); idem, Reserva de Cerrado da UNESP, 4.XII.2019, fl., *P.L.R. Moraes et al. 5539* (HRCB).

Eugenia bimarginata DC.: BRASIL. SÃO PAULO: Corumbataí, Reserva de Cerrado, 3.III.1962, fr., *n.d.* (HRCB 1457); idem, Cerrado de Corumbataí, 13.VII.1981, fr., *O. Cesar & S.N. Pagano 41* (HRCB); idem, Cerrado de Corumbataí, 26.IV.1983, bud, fl. & fr., *M.J.O. Campos 98* (HRCB); idem, Corumbataí, 7.III.1985, *M.J.O. Campos 142* (HRCB); idem, Reserva de Cerrado da UNESP, 18.V.2019, bud, *L.S. Santos et al. 49* (HRCB); idem, 12.II.2020, bud, fl. & fr., *L.S. Santos & M.M. Suemitsu 97* (HRCB); idem, 30.IX.2019, fr., *L.S. Santos & T.S. El Hindi 194* (HRCB).

Eugenia cf. *hiemalis* Cambess.: BRASIL. SÃO PAULO: Corumbataí, Reserva de Cerrado da UNESP, 18.V.2019, bud, fl., *L.S. Santos et al. 17* (HRCB); idem, 14.VIII.2019, fr., *L.S. Santos et al. 170* (HRCB); idem, 18.V.2021, bud, fl., *L.S. Santos et al. 353* (HRCB).

Eugenia punicifolia (Kunth) DC.: BRASIL. SÃO PAULO: Corumbataí, Reserva de Cerrado, 6.VII.1962, fr., *H. Amaral* (HRCB 1455); idem, Cerrado de Corumbataí, 23.V.1980, bud, fl., *O. Cesar & S.N. Pagano 44* (HRCB); idem, 7.III.1983, bud, fl., *M.J.O. Campos 144* (HRCB); idem, Corumbataí, 11.VII.1984, *C.M. Beltrati 70* (HRCB); idem, Reserva de Cerrado da UNESP, 18.V.2019, bud, fl., *L.S. Santos et al. 20* (HRCB); idem, 1.VIII.2019, fr., *L.S. Santos et al. 142* (HRCB); idem, 10.I.2020, bud, fl., *L.S. Santos & R.N. Leite 252* (HRCB); idem, 6.VII.2021, fr., *L.S. Santos et al. 347* (HRCB); idem, 18.V.2021, fr., *L.S. Santos et al. 355* (HRCB).

Myrcia bella Cambess.: BRASIL. SÃO PAULO: Corumbataí, Reserva de Cerrado, 14.III.1962, bud, fl., *n.d.* (HRCB 1453); idem, Cerrado de Corumbataí, 20.IX.1982, bud, fl., *O. Cesar* (HRCB 3176); idem, Cerrado de Corumbataí, 31.X.1983, fl., *O. Cesar* (HRCB 3542); Corumbataí, 17.V.1984, bud, fl., *S.N. Pagano 624* (HRCB); idem, Corumbataí, 15.VIII.1984, fl., *Turma Biologia* (HRCB 4664); Reserva de Cerrado da UNESP, 4.XII.2019, fr., *P.L.R. Moraes et al. 5515* (HRCB).

Myrcia guianensis (Aubl.) DC.: BRASIL. SÃO PAULO: Corumbataí, Reserva de Cerrado, 18.X.1962, bud, fl., *n.d.* (HRCB 1456); idem, Cerrado de Corumbataí, 13.VII.1981, fl., *O. Cesar & S.N. Pagano 33* (HRCB); idem, Corumbataí, 22.X.1984, bud, fl., *J.A. Mendes 22* (HRCB); idem, Cerrado de Corumbataí, n.d., bud, fl., *O. Cesar* (HRCB 3164); Reserva de Cerrado da UNESP, 18.V.2019, fr., *L.S. Santos et al. 37* (HRCB); idem, 18.V.2019, bud, fl., *L.S. Santos et al. 60* (HRCB); idem, 13.VI.2019, fr., *L.S. Santos et al. 70* (HRCB); idem, 13.VII.2019, fl. & fr., *L.S. Santos et al. 108* (HRCB); idem, 1.VIII.2019, fr., *L.S. Santos et al. 151* (HRCB); idem, 30.IX.2019, bud, fl., *L.S. Santos & T.S. El Hindi 192* (HRCB); idem, 20.XI.2020, fr., *L.S. Santos & L.P. Elias 294* (HRCB).

Myrcia neoclusiifolia A.R.Lourenço & E.Lucas: BRASIL. SÃO PAULO: Corumbataí, Reserva de Cerrado da UNESP, 29.I.2020, bud, fl., *L.S. Santos & M.N. Saka 258* (HRCB).

Myrcia splendens (Sw.) DC.: BRASIL. SÃO PAULO: Corumbataí, Reserva de Cerrado, 14.III.1961, fl., *n.d.* (HRCB 1451); Corumbataí, 7.III.1985, bud, fl., *M.J.O. Campos 124* (HRCB); idem, Reserva de Cerrado da UNESP, 11.XII.2019, bud, fl., *L.S. Santos & L.P. Elias 229* (HRCB).

Myrcia tomentosa (Aubl.) DC.: BRASIL. SÃO PAULO: Corumbataí, 18.IX.1984, bud, *S.N. Pagano 687* (HRCB); idem, 22.X.1984, fl. & fr., *J.A. Mendes 24* (HRCB).

Myrciaria floribunda (H.West ex Willd.) O.Berg: BRASIL. SÃO PAULO: Corumbataí, .IV.1983, bud, fl., *M.J.O. Campos 141* (HRCB); idem, 4.IV.1984, fl., *L. Cordeiro 7* (HRCB); idem, 17.V.1984, fl., *S.N. Pagano 634* (HRCB); idem, Reserva de Cerrado da UNESP, 13.VII.2019, fr., *L.S. Santos et al. 104* (HRCB).

Psidium grandifolium Mart. ex DC.: BRASIL. SÃO PAULO: Corumbataí, Reserva de Cerrado, 19.X.1962, fl., *H. Amaral* (HRCB 1452); idem, 15.II.1963, bud, fl., *H. Vitti* (HRCB 1449).

Nyctaginaceae

Guapira noxia (Netto) Lundell: BRASIL. SÃO PAULO: Corumbataí, Reserva de Cerrado, 10.XII.1962, fl. & fr., *H. Amaral* (HRCB 1460); Reserva de Cerrado, 20.XII.1962, fr., *J. Mattos* (HRCB 1458); idem, 15.VIII.1984, bud, fl., *A. Furlan & O. Cesar 180* (HRCB); idem, 31.VIII.1984, fl., *S.N. Pagano 619* (HRCB); idem, 19.X.1988, fl., *L.C. Saraiva 52* (HRCB); idem, 26.X.1994, fr., *Adriane 3* (HRCB); idem, Reserva da UNESP, 10.VIII.2006, fl., *J.A. Lombardi et al. 6405* (HRCB); idem, Reserva de Cerrado, n.d., bud, *H. Amaral* (HRCB 1459); Reserva de Cerrado da UNESP, 30.IX.2019, fl., *L.S. Santos & T.S. El Hindi 189* (HRCB); idem, 29.X.2020, fl., *L.S. Santos et al. 277* (HRCB); idem, 29.X.2020, fl., *L.S. Santos et al. 290* (HRCB).

Neea theifera Oerst.: BRASIL. SÃO PAULO: Corumbataí, 9.II.1984, bud, *O. Cesar 148* (HRCB); idem, 11.VII.1984, fr., *C.M. Beltrati 70* (HRCB); idem, 10.X.1985, fl., *A.R. Bueno et al.* (HRCB 7171); idem, 31.X.1991, bud, *A. Furlan 1338* (HRCB); idem, Área de Proteção Ambiental sob responsabilidade da UNESP, 24.II.2000, fr., *V.F.O. Miranda et al. 166* (HRCB); idem, Reserva de Cerrado da UNESP, 4.XII.2019, fr., *P.L.R. Moraes et al. 5517* (HRCB); idem, 11.XII.2019, fl. & fr., *L.S. Santos & L.P. Elias 232* (HRCB); idem, 26.I.2021, fr., *L.S. Santos & L.P. Elias 308* (HRCB).

Ochnaceae

Ouratea spectabilis (Mart.) Engl.: BRASIL. SÃO PAULO: Corumbataí, 26.IX.1987, bud, fl., *A. Furlan 353* (HRCB); idem, 21.V.1984, bud, fl., *S.N. Pagano 621* (HRCB); idem, 15.VIII.1984, bud, fl. & fr., *Turma Biol.* (HRCB 4535); Reserva de Cerrado da UNESP, 14.VIII.2019, bud, fl., *L.S. Santos et al. 178* (HRCB); idem, 11.XII.2019, fr., *L.S. Santos & L.P. Elias 227* (HRCB); idem, 29.X.2020, fr., *L.S. Santos et al. 289* (HRCB); idem, 4.XII.2019, fr., *P.L.R. Moraes et al. 5514* (HRCB).

Peraceae

Pera glabrata (Schott) Poepp. ex Baill.: BRASIL. SÃO PAULO: Corumbataí, Reserva de Cerrado, 20.VII.1977, fr., *O. Aulino* (HRCB 1131); Reserva de Cerrado, 1.II.1978, bud, *O. Aulino* (HRCB 1132); idem, 2.VI.1981, fr., *E.M.C. Mina* (HRCB 4530); idem, Cerrado de Corumbataí, fr., *O. Cesar & S.N. Pagano 51* (HRCB); idem, 20.IX.1982, fr., *E.M.C. Mina* (HRCB 4531); idem, Cerrado de Corumbataí, .X.1982, bud, fl., *O. Cesar* (HRCB 3156); 9.II.1984, bud, *O. Cesar 144* (HRCB); idem, 21.V.1984, fr., *J.A. Mendes 14* (HRCBR); idem, 21.V.1984, bud, *S.N. Pagano 636* (HRCB); idem, 24.II.2000, bud, *C.E. Carneiro et al. 27* (HRCB); idem, Área de Proteção Ambiental sob responsabilidade da UNESP, 24.II.2000, bud, fl., *V.F.O. Miranda et al. 171* (HRCB); idem, 24.II.2000, bud, *V.B. Ziparro et al. 1887* (HRCB); idem, 24.II.2000, bud, *V.B. Ziparro 1900* (HRCB); idem, Cerrado de Corumbataí, bud, fl., *M.H.O. Pinheiro* (HRCB 45190); Reserva de Cerrado da UNESP, 18.V.2019, bud, *L.S. Santos et al. 9* (HRCB); idem, 13.VI.2019, fl. & fr., *L.S. Santos et al. 73* (HRCB); idem, 13.VII.2019, fl. & fr., *L.S. Santos et al. 84* (HRCB); idem, 13.VII.2019, fr., *L.S. Santos et al. 121* (HRCB); idem, 1.VIII.2019, fr., *L.S. Santos et al. 127* (HRCB); idem, 12.II.2020, fl., *L.S. Santos & M.M. Suemitsu 129* (HRCB); idem, 11.III.2021, fl., *L.S. Santos et al. 323* (HRCB); idem, 18.V.2021, fr., *L.S. Santos et al. 358* (HRCB); idem, 16.VI.2021, fr., *L.S. Santos et al. 371* (HRCB); idem, 4.XII.2019, bud, *P.L.R. Moraes et al. 5543* (HRCB).

Polygalaceae

Bredemeyera floribunda Willd.: BRASIL. SÃO PAULO: Corumbataí, Reserva de Cerrado, 18.IX.1965, bud, fl., *H. Vitti* (HRCB 1480); Corumbataí, 5.III.1985, bud, fl., *L.C. Saraiva 43* (HRCB); idem, Reserva de Cerrado da UNESP, 18.V.2019, bud, *L.S. Santos et al. 54* (HRCB); idem, 29.I.2020, fl., *L.S. Santos & M.N. Saka 260* (HRCB); idem, 11.III.2021, bud, *L.S. Santos et al. 319* (HRCB).

Polygala poaya Mart.: BRASIL. SÃO PAULO: Corumbataí, Reserva de Cerrado, 15.IX.1960, *B.D. Oliveira* (HRCB 1479); idem, 10.IV.1961, *H. Vitti* (HRCB 1478); idem, 20.II.1978, *J.C. Toledo* (HRCB 1311); Corumbataí, 9.II.1984, *O. Cesar 156* (HRCB); idem, 23.IX.1987, *A. Furlan 352* (HRCB); idem, 31.X.1991, *A. Furlan 1339* (HRCB).

Primulaceae

Myrsine cf. *coriacea* (Sw.) R.Br. ex Roem. & Schult.: BRASIL. SÃO PAULO: Corumbataí, Cerrado de Corumbataí, 30.VI.1981, fr., *O. Cesar & S.N. Pagano 26* (HRCB).

Myrsine guianensis (Aubl.) Kuntze: BRASIL. SÃO PAULO: Corumbataí, Reserva de Cerrado, 23.IV.1960, fl. & fr., *H. Amaral* (HRCB 1398); idem; 15.VIII.1984, fl., *Turma-Biologia* (HRCB 4659); idem, 31.VIII.1984, fl., *S.N. Pagano 614* (HRCB); idem, 28.II.1989, fl., *L.C. Saraiva 63* (HRCB).

Myrsine lancifolia Mart.: BRASIL. SÃO PAULO: Corumbataí, 31.X.1983, fl., *O. Cesar 110* (HRCB); idem, Reserva de Cerrado da UNESP, 18.V.2019, fr., *L.S. Santos et al. 56* (HRCB).

Myrsine umbellata Mart.: BRASIL. SÃO PAULO: Corumbataí, Cerrado de Corumbataí, 30.VI.1981, fl., *O. Cesar & S.N. Pagano 39* (HRCB); idem, Reserva de Cerrado, 7.VII.1981, fl. & fr., *Depto de Botânica 263* (HRCB); idem, 12.IX.1985, fr., *S.N. Pagano 681* (HRCB). 6.VII.1989, fl., *L.C. Saraiva 67* (HRCB); idem, Reserva de Cerrado da UNESP, 1.VIII.2019, fl., *L.S. Santos et al. 132* (HRCB); idem, 1.VIII.2019, fl., *L.S. Santos et al. 145* (HRCB).

Proteaceae

Roupala montana Aubl.: BRASIL. SÃO PAULO: Corumbataí, Cerrado de Corumbataí, 25.VIII.1982, bud, *O. Cesar & S.N. Pagano 47* (HRCB); idem, Cerrado de Corumbataí, 18.VIII.1983, bud, *M.J.O. Campos 109* (HRCB); idem, 31.VIII.1984, bud, fl., *S.N. Pagano 622* (HRCB); idem, 6.VII.1989, bud, fl., *L.C. Saraiva 68* (HRCB); idem, Reserva de Cerrado da UNESP, 13.VII.2019, bud, fl., *L.S. Santos et al. 113* (HRCB); idem, 1.VIII.2019, bud, *L.S. Santos et al. 148* (HRCB); idem, 30.IX.2019, fl. & fr., *L.S. Santos & T.S. El Hindi 193* (HRCB); idem, 18.V.2021, bud, fl., *L.S. Santos et al. 350* (HRCB); idem, 6.VII.2021, bud, fl., *L.S. Santos et al. 375* (HRCB).

Rubiaceae

Amaioua intermedia Mart.: BRASIL. SÃO PAULO: Corumbataí, Reserva de Cerrado, 19.XII.1977, fr., *O. Aulino* (HRCB 1128); Reserva de Cerrado, 24.IV.1980, fr., *O. Aulino* (HRCB 1499); idem, Cerrado de Corumbataí, 21.XII.1982, fl., *M.J.O. Campos 46* (HRCB); idem, Corumbataí, 22.X.1984, bud, fl., *J.A. Mendes 19* (HRCB); idem, 3.IV.1985, fr., *A. Furlan & O. Cesar 237* (HRCB); idem, 5.XI.1985, bud, fl., *O.A. Silva* (HRCB 4762); idem, 24.II.2000, fr., *C.E. Carneiro et al. 28* (HRCB); idem, Área de Proteção ambiental sob responsabilidade da UNESP, 24.II.2000, fr., *V.F.O. Miranda et al. 165* (HRCB); idem, Reserva de Cerrado da UNESP, 11.XII.2019, fl., *L.S. Santos & L.P. Elias 30* (HRCB); idem, 14.VIII.2019, fr., *L.S. Santos et al. 161* (HRCB); idem, 4.XII.2019, bud, fl., *P.L.R. Moraes et al.* 5518 (HRCB); idem, 4.XII.2019, fl., *L.S. Santos et al. 203* (HRCB); idem, 17.XII.2019, bud, fl., *L.S. Santos & L.P. Elias 241* (HRCB); idem, 10.I.2020, fr., *L.S. Santos & R.N. Leite 256* (HRCB); idem, 8.IV.2021, fr., *L.S. Santos et al. 329* (HRCB).

Cordiera obtusa (K.Schum.) Kuntze: BRASIL. SÃO PAULO: Corumbataí, Reserva da UNESP, 10.VIII.2006, bud, fl., *J.A. Lombardi et al. 6403* (HRCB); idem, Reserva de Cerrado, 17.II.1982, bud, fl., *M.J.O. Campos 22* (HRCB).

Cordiera sessilis (Vell.) Kuntze: BRASIL. SÃO PAULO: Corumbataí, Reserva de Cerrado da UNESP, 30.IX.2019, fr., *L.S. Santos & T.S. El Hindi 200* (HRCB); idem, 4.XII.2019, fr., *P.L.R. Moraes et al.* 5540 (HRCB).

Declieuxia fruticosa (Willd. ex Roem. & Schult.) Kuntze: BRASIL. SÃO PAULO: Corumbataí, Reserva de Cerrado, 7.XII.1960, bud, fl., *B.A.D. Oliveira* (HRCB 1364).

Palicourea hoffmannseggiana (Schult.) Borhidi: Brasil: São Paulo: Corumbataí, 10.I.1983, fl. & fr., *M.J.O. Campos 72* (HRCB); idem, 27.IV.2001, fl., *C.G. Araújo* (HRCB 34028); Reserva de Cerrado da UNESP, 13.VII.2019, fr., *L.S. Santos et al. 105* (HRCB).

Palicourea marcgravii A.St.-Hil.: BRASIL. SÃO PAULO: Corumbataí, 28.XII.1983, bud, fl., *J.A. Mendes* (HRCB 3986); idem, 24.II.2000, bud, fl., *C.E. Carneiro et al. 22* (HRCB); idem, 27.IV.2001, fr., *C.G. Araújo* (HRCB 34027); Reserva de Cerrado da UNESP, 13.VII.2019, bud, *L.S. Santos et al. 94* (HRCB); idem, 14.VIII.2019, bud, fr., *L.S. Santos et al. 164* (HRCB); idem, 17.XII.2019, bud, fl., *L.S. Santos & L.P. Elias 239* (HRCB); idem, 10.I.2020, bud, fl., *L.S. Santos & R.N. Leite 250* (HRCB).

Palicourea rigida Kunth: BRASIL. SÃO PAULO: Corumbataí, Reserva de Cerrado, 29.XI.1960, bud, fl., *H. Amaral* (HRCB 1494); Reserva de Cerrado, 18.X.1961, bud, *H. Amaral* (HRCB 1495); Reserva de Cerrado,

14.VII.1964, bud, *H. Vitti* (HRCB 1491); Reserva de Cerrado, 26.VII.1964, bud, *H. Vitti* (HRCB 1496); idem, 21.XII.1982, bud, fl., *M.J.O. Campos 45* (HRCB); idem, 28.XII.1983, bud, fl., *J.A. Mendes* (HRCB 3983).

Palicourea sessilis (Vell.) C.M.Taylor: BRASIL. SÃO PAULO: Corumbataí, Cerrado de Corumbataí, 30.VI.1981, fl. & fr., *O. Cesar & S.N. Pagano 42* (HRCB); idem, Corumbataí, 26.X.1982, bud, fl., *M.J.O. Campos 37* (HRCB); idem, 25.I.1984, fr., *J.A. Mendes* (HRCB 3991); idem, Cerrado de Corumbataí, 16.X.1996, bud, fl., *A.M.R. Santos 1* (HRCB); idem, 27.IV.2001, fr., *C.G. Araújo* (HRCB 34029); idem, Reserva de Cerrado da UNESP, 18.V.2019, fr., *L.S. Santos et al. 29* (HRCB); idem, 18.V.2019, fr., *L.S. Santos et al. 47* (HRCB); idem, 4.XII.2019, bud, fl., *L.S. Santos et al. 204* (HRCB).

Palicourea violacea (Aubl.) A.Rich: BRASIL. SÃO PAULO: Corumbataí, 17.V.1984, fr., *S.N. Pagano 629* (HRCB); idem, IX.1987, bud, fl., *R. Monteiro et al.* (HRCB 8632); idem, 10.XI.1988, fl., *L.C. Saraiva 57* (HRCB); idem, 16.X.1996, fl., *A.S. Pires et al. 1* (HRCB); idem, 18.XI.1998, fr., *M.A. Assis & V.T. Rampin 1262* (HRCB); idem, Reserva de Cerrado da UNESP, 18.V.2019, fl., *L.S. Santos et al. 27* (HRCB); idem, 13.VII.2019, fr., *L.S. Santos et al. 87* (HRCB); idem, 11.IX.2019, fr., *L.S. Santos & G.G. Queiros 95* (HRCB); idem, 20.XI.2020, fl., *L.S. Santos & L.P. Elias 293* (HRCB).

Tocoyena formosa (Cham. & Schltdl.) K.Schum.: BRASIL. SÃO PAULO: Corumbataí, Reserva de Cerrado, 11.X.1960, bud, fl., *H. Amaral* (HRCB 1492); Reserva de Cerrado, 16.XI.1960, bud, fl., *H. Amaral* (HRCB 1498); idem, Cerrado de Corumbataí, 7.XII.1982, bud, fl., *M.J.O. Campos 61* (HRCB 6115); idem, 28.XII.1983, bud, fl., *J.A. Mendes* (HRCB 3985); idem, Reserva de Cerrado da UNESP, 12.II.2020, fr., *L.S. Santos & M.M. Suemitsu 165* (HRCB); idem, 4.XII.2019, bud, fl., *L.S. Santos et al. 214* (HRCB).

Salicaceae

Casearia sylvestris Sw.: BRASIL. SÃO PAULO: Corumbataí, Reserva de Cerrado, 15.IV.1961, bud, fl., *H. Amaral* (HRCB 1282); Reserva de Cerrado, 18.VIII.1961, bud, fl., *H. Amaral* (HRCB 1281); Reserva de Cerrado, 28.II.1963, bud, fl., *H. Vitti* (HRCB 1284); idem, 16.IX.1966, bud, fl., *H. Vitti* (HRCB 1285); idem, 31.VIII.1984, fl., *S.N. Pagano 618* (HRCB); idem, 20.IV.1985, fl., *M.J.O. Campos 140* (HRCB); idem, Reserva de Cerrado, n.d., bud, fl., *n.d.* (HRCB 1283); Reserva de Cerrado da UNESP, 11.IX.2019, fr., *L.S. Santos & G.G. Queiros 22* (HRCB); idem, 14.VIII.2019, bud, fl., *L.S. Santos et al. 173* (HRCB); idem, 14.VIII.2019, bud, fl., *L.S. Santos et al. 177* (HRCB); idem, 30.IX.2019, fl. & fr., *L.S. Santos & T.S. El Hindi 186* (HRCB); idem, 16.VI.2021, bud, fl., *L.S. Santos et al. 363* (HRCB).

Sapindaceae

Serjania erecta Radlk.: BRASIL. SÃO PAULO: Corumbataí, Reserva de Cerrado, 6.XII.1965, *H. Vitti* (HRCB 1589); Corumbataí, 21.II.1982, *M.J.O. Campos* 49 (HRCB).

Talisia angustifolia Radlk.: BRASIL. SÃO PAULO: Corumbataí, Reserva de Cerrado, 6.II.1963, fl. & fr., *H. Vitti* (HRCB 1587).

Sapotaceae

Pouteria ramiflora (Mart.) Radlk.: BRASIL. SÃO PAULO: Corumbataí, 12.IX.1985, bud, fl., *S.N. Pagano 694* (HRCB).

Pouteria torta (Mart.) Radlk.: BRASIL. SÃO PAULO: Corumbataí, Reserva de Cerrado, 11.IX.1962, bud, *H. Amaral* (HRCB 1590); idem, Cerrado de Corumbataí, 24.VIII.1981, bud, fl., *O. Cesar & S.N. Pagano 58* (HRCB); idem, 4.VII.1989, bud, *L.C. Saraiva 65* (HRCB).

Siparunaceae

Siparuna guianensis Aubl.: BRASIL. SÃO PAULO: Corumbataí, 31.VIII.1984, fl., *S.N. Pagano 615* (HRCB); idem, 22.X.1984, fr., *J.A. Mendes 21* (HRCB); idem, 24.II.2000, fr., *C.E. Carneiro et al. 32* (HRCB); idem, Área de Proteção ambiental sob responsabilidade da UNESP, 24.II.2000, fr., *V.F.O. Miranda et al. 170* (HRCB); idem, Reserva de Cerrado da UNESP, 18.V.2019, fr., *L.S. Santos et al. 14* (HRCB); idem, 13.VII.2019, bud, fr., *L.S. Santos et al. 92* (HRCB); idem, 8.IV.2021, fr., *L.S. Santos et al. 330* (HRCB).

Solanaceae

Cestrum mariquitense Kunth: BRASIL. SÃO PAULO: Corumbataí, Reserva de Cerrado, 28.VI.1962, fl., *H. Amaral* (HRCB 1601); idem, Cerrado de Corumbataí, 3.X.1983, fl. & fr., *O. Cesar* (HRCB 3503); Reserva de Cerrado, n.d., fl., *n.d.* (HRCB 1603); idem, Reserva de Cerrado da UNESP, 1.VIII.2019, fl., *L.S. Santos et al. 130* (HRCB); idem, 29.I.2020, fl., *L.S. Santos & M.N. Saka 257* (HRCB).

Solanum cf. *didymum* Dunal: BRASIL. SÃO PAULO: Corumbataí, Reserva de Cerrado da UNESP, 8.IV.2021, bud, fl., *L.S. Santos et al. 335* (HRCB).

Solanum granulosoleprosum Dunal: BRASIL. SÃO PAULO: Corumbataí, 9.II.1984, bud, fl. & fr., *O. Cesar 141* (HRCB); idem, Reserva de Cerrado da UNESP, 18.V.2019, fl. & fr., *L.S. Santos et al. 36* (HRCB); idem, 12.II.2020, bud, fl., *L.S. Santos & M.M. Suemitsu 111* (HRCB); idem, 29.I.2020, fl., *L.S. Santos & M.N. Saka 269* (HRCB).

Solanum lycocarpum A.St.-Hil.: BRASIL. SÃO PAULO: Corumbataí, Reserva de Cerrado, 29.III.1976, bud, fl., *G. Marinis & O. Cesar* (HRCB 1602); idem, 10.IX.1982, bud, fl., *M.J.O. Campos 3* (HRCB); idem, Reserva de Cerrado, 11.V.1983, bud, fl., *L.C. Saraiva 20* (HRCB); idem, 25.I.1984, bud, fl., *J.A. Mendes* (HRCB 3990); idem, 15.VIII.1984, bud, fl., *Turma-Biologia* (HRCB 4655); Reserva de Cerrado da UNESP, 14.VIII.2019, fr., *L.S. Santos et al. 175* (HRCB); idem, 30.IX.2019, bud, fl., *L.S. Santos & T.S. El Hindi 188* (HRCB); idem, 29.X.2020, bud, fl., *L.S. Santos et al. 274* (HRCB); idem, 26.I.2021, bud, fl., *L.S. Santos & L.P. Elias 298* (HRCB).

Styracaceae

Styrax camporum Pohl: BRASIL. SÃO PAULO: Corumbataí, Cerrado de Corumbataí, 10.VIII.1981, bud, fl., *O. Cesar & S.N. Pagano 43* (HRCB); idem, Cerrado de Corumbataí, 28.VI.1983, bud, fl., *L.C. Saraiva 28* (HRCB); idem, C, bud, fl., *M.J.O. Campos 40* (HRCB); idem, 25.I.1984, bud, fl., *J.A. Mendes* (HRCB 3987); idem, 31.VIII.1984, fr., *S.N. Pagano 607* (HRCB); idem, 24.II.2000, fl. & fr., *V.B. Ziparro et al. 1888* (HRCB); idem, 12.IV.2013, bud, fl. & fr., *A.C.G. Bressan 2* (HRCB); idem, Reserva de Cerrado da UNESP, 18.V.2019, bud, fl. & fr., *L.S. Santos et al. 39* (HRCB)13.VI.2019, bud, fl. & fr., *L.S. Santos et al. 72* (HRCB); idem, 10.I.2020, bud, fl., *L.S. Santos & R.N. Leite 249* (HRCB); idem, 26.I.2021, bud, fl. & fr., *L.S. Santos & L.P. Elias 314* (HRCB); idem, 18.V.2021, bud, fl., *L.S. Santos et al. 351* (HRCB); idem, 16.VI.2021, fr., *L.S. Santos et al. 368* (HRCB).

Styrax ferrugineus Nees & Mart.: BRASIL. SÃO PAULO: Corumbataí, Reserva de Cerrado, 10.IV.1964, bud, fl., *H. Vitti* (1596); Reserva de Cerrado, 18.V.1977, bud, fl., *G. Marinis & O. Cesar 23* (HRCB); idem, Cerrado de Corumbataí, 30.VI.1981, bud, *O. Cesar & S.N. Pagano 19* (HRCB); idem, Cerrado de Corumbataí, 28.VI.1983, bud, fl., *L.C. Saraiva 27* (HRCB).

Symplocaceae

Symplocos oblongifolia Casar.: BRASIL. SÃO PAULO: Corumbataí, Cerrado de Corumbataí, 7.VII.1981, bud, fl., *O. Cesar & S.N. Pagano 22* (HRCB); idem, 13.VII.1981, bud, *O. Cesar & S.N. Pagano 40* (HRCB). Corumbataí, 16.VI.1988, bud, fl., *L.C. Saraiva 48* (HRCB).

Symplocos pubescens Klotzsch ex Benth.: BRASIL. SÃO PAULO: Corumbataí, 9.II.1984, fl., *O. Cesar 145* (HRCB); idem, 16.VI.1988, fl. & fr., *L.C. Saraiva* (HRCB 8875); idem, 16.VI.1988, bud, fl. & fr., *L.C. Saraiva 47* (HRCB); idem, 1.XII.1999, bud, fl., *M.A. Assis & V.T. Rampin 1330* (HRCB); idem, Reserva de Cerrado da UNESP, 18.V.2019, fr., *L.S. Santos et al. 3* (HRCB).

Thymelaeaceae

Daphnopsis fasciculata (Meisn.) Nevling: BRASIL. SÃO PAULO: Corumbataí, 18.IX.1984, *S.N. Pagano 688* (HRCB); idem, Reserva de Cerrado, 13.II.1989, fl., *L.C. Saraiva 62* (HRCB); idem, II.1996, fl., *V.T. Rampin 833* (HRCB); idem, 2.XII.1997, fl., *M.A. Assis & V.T. Rampin 1077* (HRCB); idem, Cerrado de Corumbataí, 30.VI.2003, fl., *M.H.O. Pinheiro* (HRCB 45191).

Verbenaceae

Lantana camara L.: BRASIL. SÃO PAULO: Corumbataí, Reserva de Cerrado da UNESP, 4.XII.2019, fl. & fr., *L.S. Santos et al. 205* (HRCB); idem, 4.XII.2019, fl. & fr., *P.L.R. Moraes et al. 5521* (HRCB).

Lantana fucata Lindl.: BRASIL. SÃO PAULO: Corumbataí, 20.II.1987, fl., *N.M.L. Cunha 10* (HRCB); idem, Reserva de Cerrado da UNESP, 1.VIII.2019, fl., *L.S. Santos et al. 128* (HRCB).

Lippia origanoides Kunth: BRASIL. SÃO PAULO: Corumbataí, 21.XII.1982, bud, fl., *M.J.O. Campos 56* (HRCB); idem, 20.II.1987, bud, fl., *N.M.L. Cunha 11* (HRCB); idem, Reserva de Cerrado da UNESP, 18.V.2019, fr., *L.S. Santos et al. 11* (HRCB); idem, 12.II.2020, fl., *L.S. Santos & M.M. Suemitsu 174* (HRCB); idem, 11.III.2021, fl., *L.S. Santos et al. 321* (HRCB).

Stachytarpheta cayennensis (Rich.) Vahl: BRASIL. SÃO PAULO: Corumbataí, Cerrado de Corumbataí, 9.XI.1982, *M.J.O. Campos 44* (HRCB).

Vochysiaceae

Qualea cordata Spreng.: BRASIL. SÃO PAULO: Corumbataí, Reserva de Cerrado, 19.VIII.1961, bud, fl., fr, *H. Amaral* (HRCB 1618); Reserva de Cerrado da UNESP, 12.III.2020, fr., *L.S. Santos & G.G. Queiros 181* (HRCB); idem, 30.IX.2019, bud, fl. & fr., *L.S. Santos & T.S. El Hindi 198* (HRCB).

Qualea grandiflora Mart.: BRASIL. SÃO PAULO: Corumbataí, Reserva de Cerrado, 29.XI.1960, bud, fl., *H. Amaral* (HRCB 1622); idem, 29.XI.1961, fl., *H. Amaral* (HRCB 1623); bud, fl., *n.d.* (HRCB 1620); idem, Reserva de Cerrado da UNESP, 1.VIII.2019, fr., *L.S. Santos et al. 141* (HRCB); idem, 4.XII.2019, bud, fl., *L.S. Santos et al. 212* (HRCB); idem, 4.XII.2019, bud, fl., *P.L.R. Moraes et al.* 5523 (HRCB); idem, 4.XII.2019, bud, fl., *P.L.R. Moraes et al. 5530* (HRCB).

Qualea multiflora Mart.: BRASIL. SÃO PAULO: Corumbataí, Reserva de Cerrado, 19.XII.1977, bud, fr., *O. Aulino* (HRCB 1129); idem, 19.XII.1977, bud, fl. & fr., *O.A. Silva* (HRCB 1624); Corumbataí, 22.X.1984, bud, *J.A. Mendes 20* (HRCB); idem, Reserva de Cerrado da UNESP, 14.VIII.2019, fr., *L.S. Santos et al. 180* (HRCB); idem, 12.II.2020, fr., *L.S. Santos & M.M. Suemitsu 197* (HRCB); idem, 11.XII.2019, bud, fl., *L.S. Santos & L.P. Elias 226* (HRCB); idem, 12.III.2020, fr., *L.S. Santos & G.G. Queiros 273* (HRCB); idem, 26.I.2021, bud, fl., *L.S. Santos & L.P. Elias 305* (HRCB).

Vochysia tucanorum Mart.: BRASIL. SÃO PAULO: Corumbataí, Reserva de Cerrado, 3.III.1966, bud, fl., *H. Vitti* (HRCB 1619); Reserva de Cerrado, 19.XII.1977, bud, fl., *O.A. Silva* (HRCB 1621); Reserva de Cerrado, 19.XII.1977, bud, fl., *O. Aulino* (HRCB 1126); idem, 25.I.1984, bud, *J.A. Mendes* (HRCB 4000); idem, 17.V.1984, fr., *S.N. Pagano 627* (HRCB); idem, Área de proteção ambiental sob responsabilidade da UNESP, 24.II.2000, fl., *V.F.O. Miranda et al. 168* (HRCB); idem, 24.II.2000, fl. & fr., *V.B. Ziparro et al. 1883* (HRCB); idem, 24.II.2000, fl., *C.E. Carneiro et al. 29* (HRBC); idem, 24.II.2000, fl., *C.E. Carneiro et al. 30* (HRCB); idem, Reserva da UNESP, 10.VIII.2006, fr., *J.A. Lombardi et al. 6408* (HRCB); idem, Reserva de Cerrado da UNESP, 18.V.2019, fl. & fr., *L.S. Santos et al. 10* (HRCB); idem, 13.VI.2019, fr., *L.S. Santos et al. 79* (HRCB); idem, 11.XII.2019, bud, fl., *L.S. Santos & L.P. Elias 225* (HRCB); idem, 26.I.2021, bud, fl., *L.S. Santos & L.P. Elias 310* (HRCB); idem, 8.IV.2021, fr., *L.S. Santos et al. 331* (HRCB); idem, 16.VI.2021, fr., *L.S. Santos et al. 367* (HRCB).
